# Context-aware synthetic promoter design using neural networks enables rewiring of eukaryotic transcriptional networks

**DOI:** 10.1038/s41540-026-00684-5

**Published:** 2026-03-17

**Authors:** Lukas Kuhajda, Tomas Honzik, Jan Svec, Daniel Georgiev

**Affiliations:** 1 New Technologies for the Information Society, University of West Bohemia in Pilsen, Pilsen, Czechia; 2 Department of Cybernetics, University of West Bohemia in Pilsen, Pilsen, Czechia

**Keywords:** Biotechnology, Computational biology and bioinformatics

## Abstract

Gene regulation through promoter engineering is a cornerstone of synthetic biology, enabling precise control over transcriptional networks. However, experimental approaches remain labor-intensive. While artificial neural networks (ANNs) have improved regulatory element prediction, tools for promoter–transcription factor binding site (TFBS) recombination are still lacking. We present an ANN framework for context-aware design of synthetic promoters in *Saccharomyces cerevisiae*. The model predicts optimal TFBS insertion sites and the extent of promoter rewriting needed for successful integration. Applying this, we screened 6,011 native yeast promoters for compatibility with the TetR TFBS, generating a ranked list of high-confidence promoter–TFBS pairs. Experimental validation showed that model-designed promoters achieved repression rates up to 98.4%, without prior experimental characterization or tuning. We further rewired the yeast transcriptional network by introducing glucose-dependent regulation of an essential gene via Mig1 TFBS insertion. These results establish a scalable, predictive method for engineering regulatory sequences and reprogramming transcriptional logic.

## Introduction

Cells regulate gene expression through complex networks of proteins, RNA molecules, and DNA sequences^1^. These regulatory systems allow organisms to respond to environmental changes, coordinate functions, and develop adaptive or novel traits. Rewiring these transcriptional networks by modifying regulatory elements can enable entirely new cellular behaviors and is a key goal in synthetic biology^2–4^.

Rewiring of transcriptional networks largely relies on the use of synthetic promoter libraries that lack the context-dependence of native promoters. Over the years, various synthetic promoter libraries have been developed to control expression strength or make promoters responsive to specific inputs by inserting transcription factor binding sites (TFBSs)^5–11^. These engineered promoters have been applied in metabolic engineering^5,12–14^, biosensors, and regulatory devices like toggle switches, logic gates, and oscillators^14–18^. Native promoters exhibit context-dependent behavior influenced by environmental conditions and genetic background. This sensitivity is retained when promoters serve as scaffolds for TFBS insertions, allowing for the construction of dynamically regulated synthetic promoters^19–23^.

Despite advances in DNA editing and assembly^24–26^, promoter engineering remains difficult due to the lack of conserved structures to guide insertion and deletion of regulatory sequences. Artificial neural networks (ANNs) have shown strong potential for resolving regulatory sequences and guiding design. Several models have been used to predict promoter strength, enhancer activity, or chromatin accessibility (Basset^27^, DanQ^28^, Basenji^29^). Other models focus on enhancer-promoter interactions (SPEID^30^, DeepSEM^31^, Enformer^32^) or a promoter strength prediction from experimental data with subsequent generative models to design promoters with specific strength^33–39^. However, current tools do not predict potential recombinations of promoters and TFBSs, a critical step for building context-aware, responsive regulatory elements.

We developed a two-stage ANN system that uses context-aware sequence recombination to propose general TFBS insertion. The first model (*Place-Back*) identifies the most suitable insertion region, and the second model (*Determiner*) refines the predicted rewrite length to ensure a good fit. The models are trained using a self-supervised approach, allowing them to learn the context of TFBS insertions directly from genomic data without any labels.

We applied the system to screen 6011 native yeast promoters for compatibility with the TetR TFBS (tetO), generating a ranked list of high-confidence promoter-TFBS pairs.

In addition, we validated two unique promoter systems in vivo: (1) untested wildtype *Saccharomyces cerevisiae* promoters were regulated by the TetR repressing system, achieving strong conditional repression up to 98.4% with no prior experimental characterization and tuning, and (2) the *S. cerevisiae* wildtype transcriptional regulatory network was rewired to create a completely new glucose sensitive response achieved through glucose-responsive regulation of an essential gene. Together, these results show that context-aware promoter design using neural networks can streamline synthetic promoter construction and enable more complex regulatory systems.

The developed tool is available as an online browser-based application and as a locally executable version via the associated GitHub repository. Access details are provided in the “Code Availability” section.

## Results

### Dataset

The target organism for this study is *S. cerevisiae*. To train an ANN with evolutionarily relevant sequences, we extracted promoter sequences for all annotated genes from 25 *Saccharomycotina* species. These sequences were split into training and validation datasets, while *S. cerevisiae* promoters were excluded and reserved solely for testing. Promoters were defined as 400 bp upstream of the respective start codon, ensuring at least 100 bp separation from the nearest neighboring gene in the promoter direction. This resulted in a dataset of 6011 *S. cerevisiae* promoters for testing, and 133,889 promoters from *Saccharomycotina,* further divided into a training dataset with 123,889 promoters, and a validation dataset with 10,000 promoters. All sequences were stored in nucleotide form for model training and evaluation.

### Two-stage model architecture for TFBS insertion

To support context-aware promoter engineering, we built a two-stage ANN system made up of two models. The first model, called *Place-Back*, predicts the most suitable site for inserting a TFBS into a given promoter sequence with two variants trained, differing in the *Model-Specific Rewriting Length* (MSRL): one replaces 5 bp (closely modeling actual TFBS insertion) and the other 40 bp (modeling larger-scale sequence replacement). The second model, called *Determiner*, then decides how much of the promoter should be overwritten, either a short (5 bp) or long (40 bp) region, and identifies the most likely region of insertion. The two models are described in detail below.

**Fig. 1:**
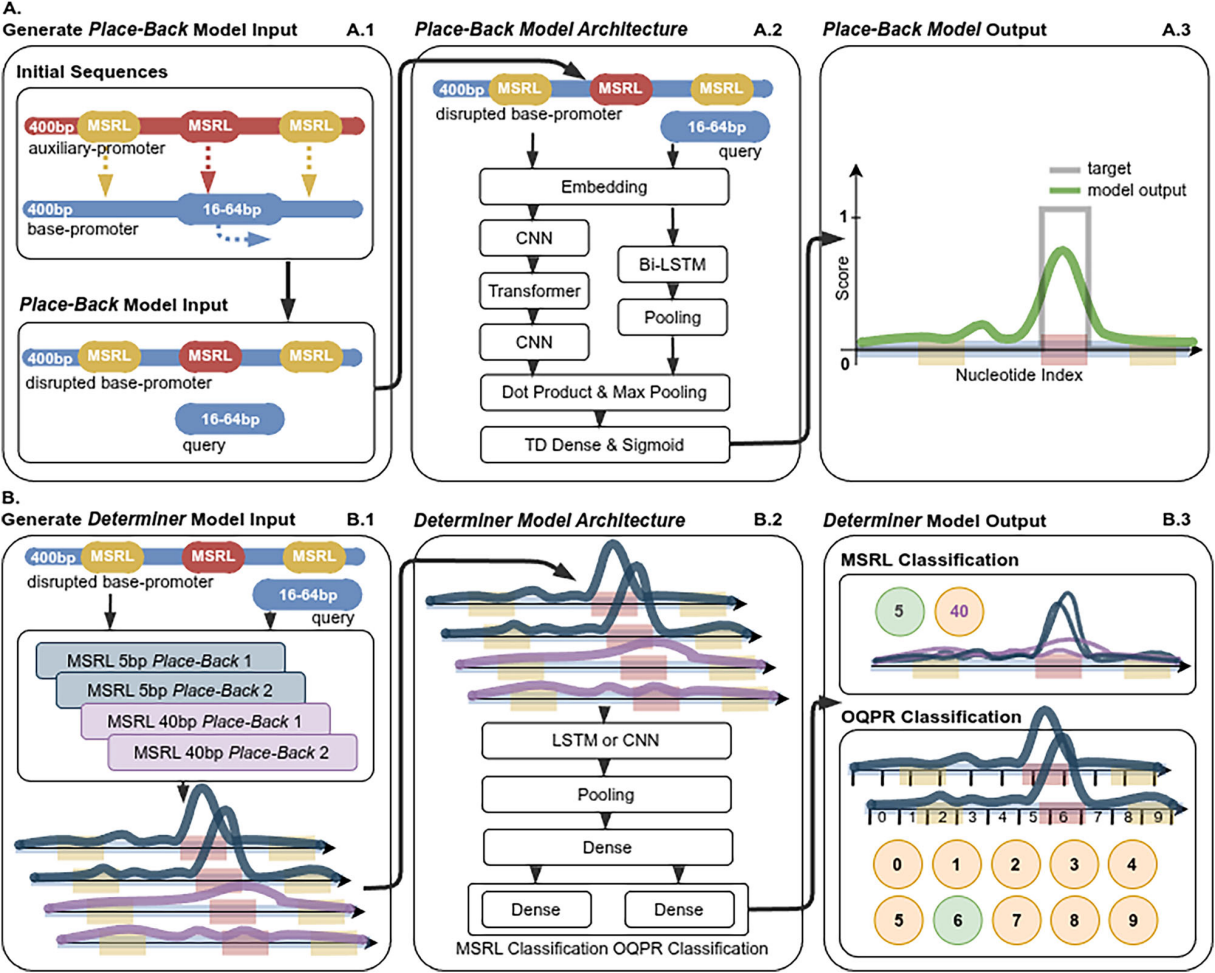
Architecture and training of the two-stage ANN system for TFBS insertion. **A Place-Back model development:** (A.1) **Training data generation for Place-Back models:** Two 400 bp promoter sequences are randomly selected, *base-promoter* and *auxiliary-promoter*. A 16–64 bp segment is extracted from the *base-promoter* (query) and replaced with a 5 bp or 40 bp (MSRL) segment from the other (*auxiliary-promoter*). To prevent trivial learning, 2–3 additional disruptions of fixed length (MSRL) are introduced at random positions. (A.2) **Place-Back model architecture:** The disrupted *base-promoter* is processed through embedding, CNNs for compression, a transformer layer, and CNNs for decompression. The query is processed via embedding and Bi-LSTM. The two branches are combined with a dot-product operation, followed by max pooling and a sigmoid output over all 400 promoter positions. (A.3) **Model output:** The resulting prediction is a vector of probabilities (0–1) across the promoter, with peaks indicating the predicted probability of the original query location within the *base-promoter*. The model is trained to assign values approaching 1 at the query’s original location and 0 elsewhere. **B Determiner model development:** (B.1) **Input to Determiner models:** A single disrupted *base-promoter* and query sequence pair is passed through four *Place-Back* models (two for MSRL = 5 bp, two for MSRL = 40 bp), generating four prediction curves. (B.2) **Determiner model architecture:** The stacked *Place-Back* outputs are processed through a CNN or LSTM-based model, followed by dense layers. (B.3) **Determiner output:** The model predicts (1) the correct rewrite length (5 bp or 40 bp) and (2) the most likely insertion region (divided into 10 bins of 40 bp each).

The *Place-Back* model uses a self-supervised training approach. To prepare a training sample (Fig. 1A.1), two promoters (400 bp each) from the same yeast species are randomly selected: *base-promoter* and *auxiliary-promoter*. A segment of 8–64 bp is extracted from the *base-promoter* to serve as the query sequence, and this query is replaced in its original position by an MSRL-length segment from the *auxiliary-promoter*. To prevent the model from simply detecting the query region by alignment, we also introduce at random positions 2–3 additional disruptions (segments of MSRL length) to the *base-promoter* from the *auxiliary-promoter*. This forces the model to learn contextual features rather than relying on exact sequence matching. To ensure robust training, query lengths (8–64 bp), query target positions, and disruption sites were sampled uniformly to cover the promoter space (distributions provided in Supplementary Figs. 1–5). *Place-Back* model takes the disrupted *base-promoter* and query as input and outputs a 400-length prediction vector indicating the probability that each base is part of the original query region. The model consists of an embedding layer with positional encoding for nucleotide representation, convolutional neural networks (CNNs) to reduce promoter length and capture local motifs, a transformer encoder to learn long-range dependencies, a bi-directional LSTM to process query sequence, a dot-product based attention mechanism to combine both inputs, and a final sigmoid activation to generate base-pair level probabilities (Fig. 1A.2,3). Two *Place-Back* models with MSRL of 5 bp and two models with MSRL of 40 bp were trained. Each model contains ~6 million parameters and was trained for 168 h on a GeForce GTX 1080 Ti GPU, processing roughly 300 million training examples.

**Fig. 2:**
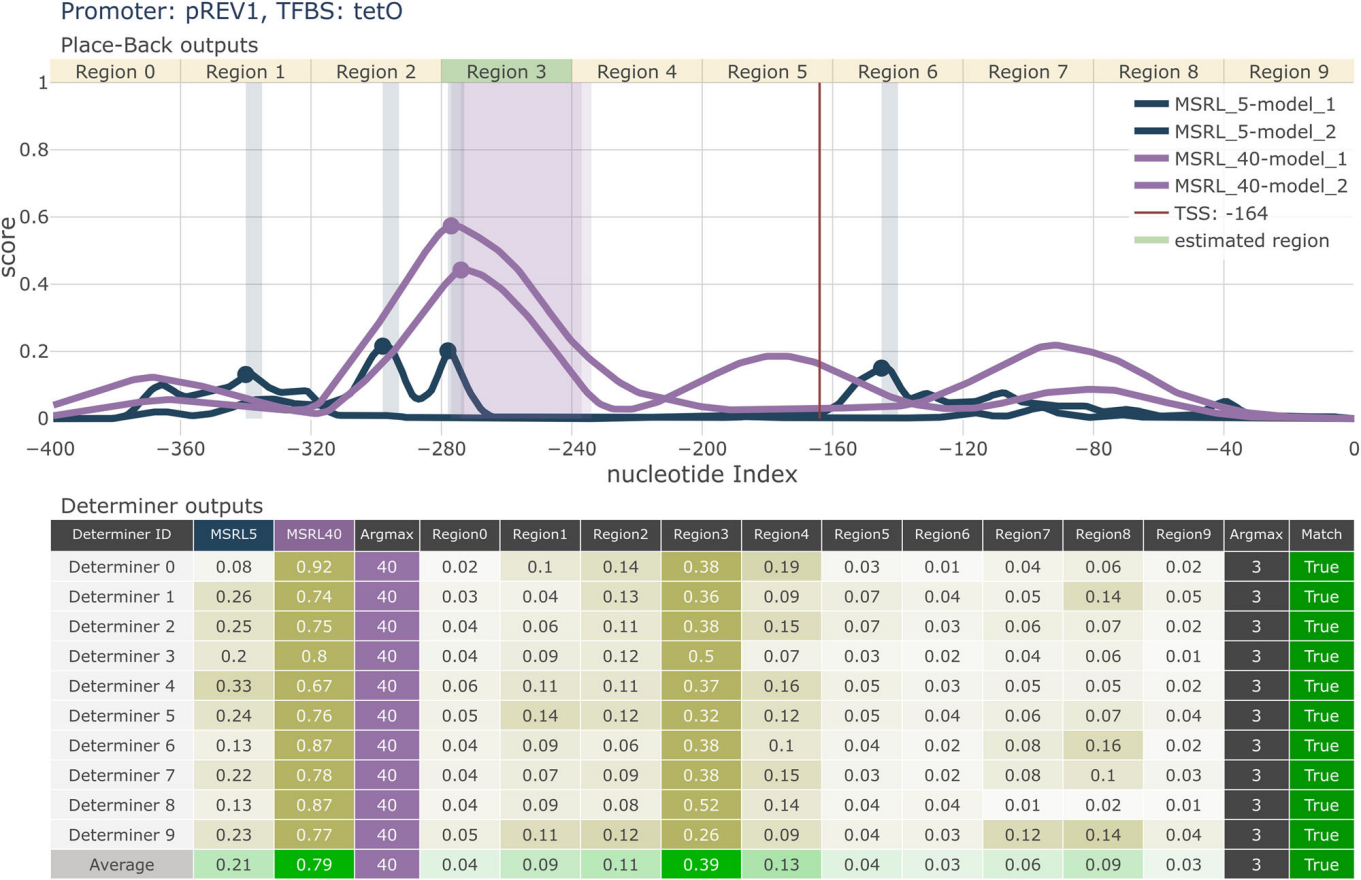
Example output from the two-stage ANN system for tetO recombination into the pREV1 promoter. Top panel: Outputs from four *Place-Back* models. Two 5 bp models (blue) and two 40 bp models (purple) show insertion probability across the promoter sequence. The red line marks the transcription start site (TSS). Bottom panel: Predictions from ten *Determiner* models. All *Determiner* models select MSRL = 40 bp and region 3 as the optimal insertion window. The highlighted region in the top panel corresponds to this classification, confirming a high-confidence recombination proposal. Based on the outputs, tetO would be rewritten to the promoter region from −277 to −237.

Outputs from the *Place-Back* models are passed to the *Determiner* model (Fig. 1B.1), which resolves inconsistencies between predictions and classifies the most likely insertion region and required rewrite length. For training, the *Determiner* receives the output curves from two 5 bp and two 40 bp *Place-Back* models for a single input sample generated with either MSRL of 5 bp or 40 bp. The *Determiner* predicts the rewrite length the sample was generated with, and the *Original Query Position Region* (OQPR)—one of ten 40 bp regions spanning the promoter (Fig. 1B.3). Ten *Determiner* models with diverse CNN or LSTM architectures (1.8–13.6 million parameters) were trained. Each *Determiner* model generates a decision based on whether the combination of its two classification outputs and the *Place-Back* outputs is internally consistent, defined by the presence of a significant peak from the selected MSRL *Place-Back* model within the specified region. Such outcomes are labeled as a “Match” in the output figures (Fig. 2). The primary system decision and corresponding experimental proposal are derived from the average prediction across all *Determiner* models. To enhance reliability, only cases in which at least 7 out of 10 *Determiner* models yielded a “Match” were classified as high-confidence predictions that are recommended for laboratory experiment (Supplementary Fig. 6).

### Model performance and prediction filtering

Model performance was evaluated on held-out validation data. The *Place-Back* models reached an average precision (accuracy of positive predictions) of 48%, meaning that nearly half of the positive predictions (top-scoring insertion locations) matched the original query placement. Recall (coverage of actual positives) values depended on the MSRL: 5 bp models had lower recall (~4%) due to narrower and lower prediction peak values, while 40 bp models had higher recall (~32%).

While these precision and recall values may appear modest by conventional machine learning standards, they are both statistically meaningful and biologically actionable in this context. The task involves locating a functional insertion site within a 400 bp promoter sequence, an inherently difficult and imbalanced classification problem. Even moderate precision, therefore, represents a significant enrichment over random chance. To quantify this enrichment, we compared the model’s performance against a baseline of random insertion. We generated 100,000 samples with random placements (avoiding TSS and TATA-box regions) in *S. cerevisiae* promoters. While our model achieved 48% precision, the random baseline yielded an expected precision of only 10.95% for MSRL 40 and 1.26% for MSRL 5. This substantial increase demonstrates that the ANN successfully captures contextual constraints well beyond simple heuristic rules.

To verify that the model generalizes to novel contexts rather than memorizing conserved sequences, we analyzed the homology between the *S. cerevisiae* test set and the training data. We found that the model’s performance was consistent regardless of whether a promoter had significant sequence similarity to the training set or not. Crucially, this independence from memorization was confirmed in vivo: our experimental validation included successful designs for both highly conserved promoters (pPAB1) and those with no or low detectable homology to the training database (pHHF2, pPOP6, pREV1, and pPCF11). This indicates the model relies on learned contextual features rather than homologous sequence retrieval.

Importantly, promoter functionality in vivo is often tolerant to slight positional variation, meaning that predictions considered incorrect by strict matching criteria may still result in successful TFBS integration. This is supported by the strong experimental performance of selected model predictions, described in the sections below.

To further increase the reliability of predictions, we introduced a filtering step based on multi-model consensus using the *Determiner*. Only predictions with strong internal consistency across multiple models were retained. This strategy provides a practical interface for experimental selection.

### In silico recombination of yeast promoters with tetO

In an inference mode, the TFBS with length 8–64 bp enters the system as the query sequence, and the promoter enters in wild-type form. An example of the overall output of the model for recombination of a wild-type promoter with a TFBS is shown in Fig. 2.

To evaluate the potential of the model in a real use case, we applied it to all 6011 *S. cerevisiae* promoters (defined as 400 bp upstream of the start codon). Each promoter was virtually recombined with the TetR TFBS (tetO: TCCCTATCAGTGATAGAGATCTCCCTATCAGTGATAGAGA^40^). The model evaluated whether tetO could be inserted into each promoter without disrupting essential elements like the TATA-box or transcription start site (TSS). Of the 6011 promoters, 3712 were recommended for recombination, while 2299 were filtered out due to a lack of model consensus or predicted disruption of minimal regulatory elements.

Among the recommended promoters, 2149 were assigned to use a 5 bp rewrite, and 1560 were assigned to use a 40 bp rewrite. Analysis of the predicted insertion sites showed that, for 5 bp modifications, 85% occurred in the distal half of the promoter, and 60% were within the last 100 bp upstream of the start codon (Supplementary Fig. 7). The 5 bp insertion sites did not display a consistent positional relationship with the TATA-box (Supplementary Fig. 8), and were generally located farther from known TSS positions (Supplementary Fig. 9). For 40 bp rewrites, about 50% of insertions also occurred in this downstream region (Supplementary Fig. 10). When TATA-box or TSS positions were known, a notable fraction of insertions occurred within 30 bp of the TATA-box (25%) or within 33 bp of the TSS (20%) (Supplementary Figs. 11–12). These patterns suggest the model prefers insertions near core regulatory regions but avoids direct disruption. A full list of promoter-TFBS pairs and insertion details is available in the Supplementary Information.

### Experimental validation of synthetic promoters with TetR

To experimentally validate the ANN predictions, we selected four *S. cerevisiae* constitutive promoters of different strengths (pHHF2, pPAB1, pPOP6, pREV1) predicted to be compatible with tetO insertion, for experimental testing. Each promoter was recombined with tetO at the position suggested by the model, either a 5 bp or 40 bp rewrite (system output with position selection shown in Supplementary Fig. 13–16), and fused to a NanoLuc reporter.

This system was orthogonal: the TetR repressor was introduced on a separate expression cassette (pCCW12-TetR), and TetR is not natively present in yeast. This means the repression system could be tested without interference from endogenous transcription factors, allowing clean validation of the ANN-designed promoter-TFBS combinations.

**Fig. 3:**
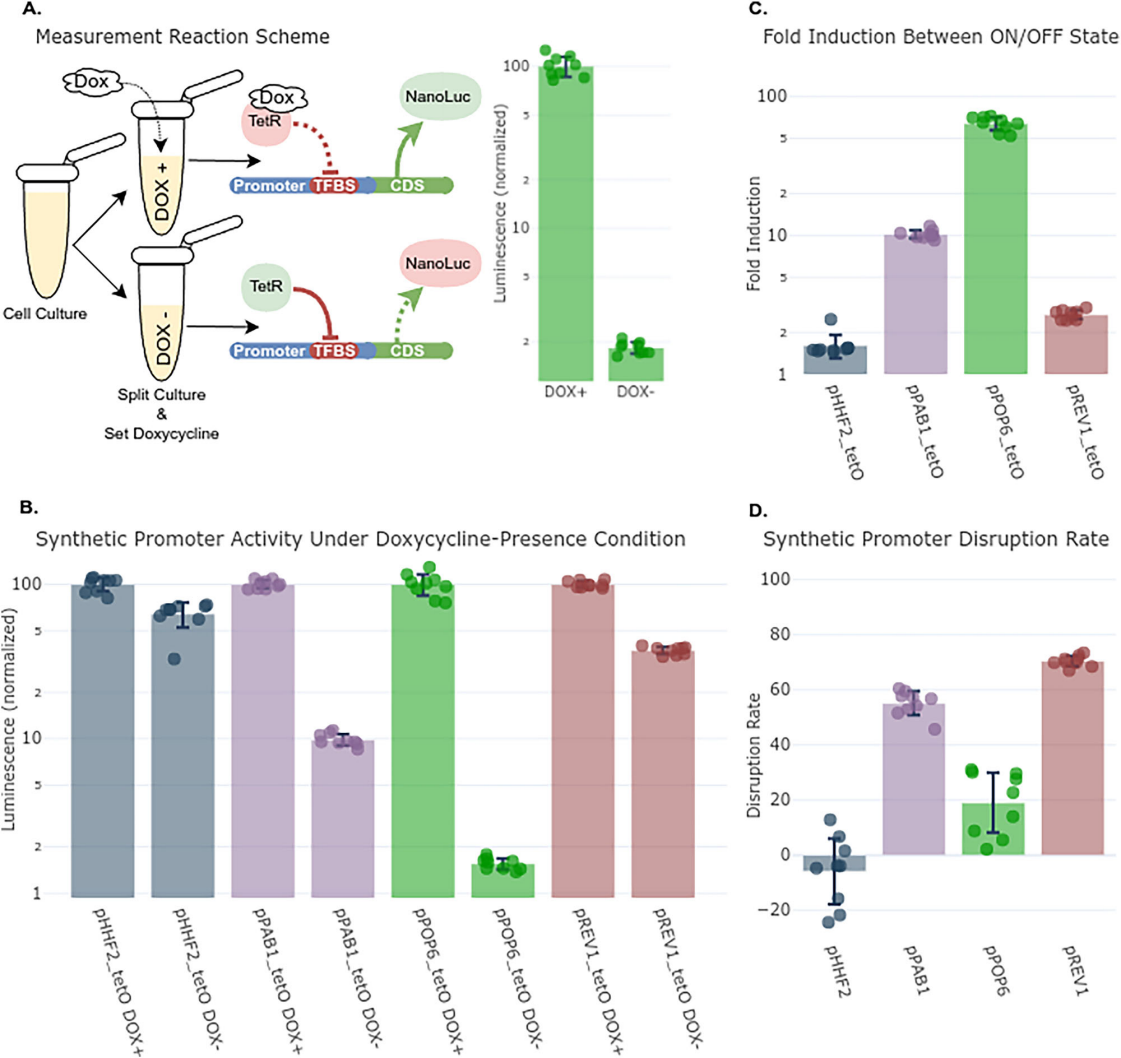
Experimental validation of ANN-designed synthetic promoters recombined with tetO and tested using an orthogonal TetR repression system. **A** **Experimental setup**: Synthetic promoters were fused to NanoLuc and integrated into yeast strains with or without TetR expression. Promoter activity was measured in ON (doxycycline present) and OFF (TetR active) states. **B** **Repression levels** of each synthetic promoter normalized to ON-state activity (100%). pPOP6_tetO showed the strongest repression (98.4%), followed by pPAB1_tetO (90.1%), pREV1_tetO (62.7%), and pHHF2_tetO (35.8%). **C** **Fold induction**: pPOP6_tetO exhibited a 64-fold ON/OFF induction, followed by pPAB1_tetO (10.2-fold), pREV1_tetO (2.7-fold), and pHHF2_tetO (1.6-fold). **D** **Impact on native activity**: Comparison of wild-type vs. synthetic promoter activity in the absence of TetR. pHHF2 showed no disruption, pPOP6 activity dropped by 19%, while pPAB1 and pREV1 exhibited 55 and 70% activity loss, respectively.

The synthetic promoters were tested in strains with and without TetR expression, with and without doxycycline induction to set deactivation of repression (Fig. 3.A). All four synthetic promoters showed strong repression in the OFF state (TetR expressed, no doxycycline), confirming the function of the model-designed recombination. Repression rates were: pPOP6_tetO - 98.4% (64.1-fold induction), pPAB1_tetO - 90.1% (10.2-fold), pREV1_tetO - 62.7% (2.7-fold), and pHHF2_tetO - 35.8% (1.6-fold) (Fig. 3.B, C). These results demonstrate that the model can identify insertion sites that enable high dynamic range regulation without manual tuning.

We also evaluated how tetO insertion affected native promoter strength by comparing wild-type vs. synthetic versions in strains lacking TetR (Fig. 3.D). Some promoters retained most of their activity (pHHF2 was unaffected, pPOP6 dropped 19%), while others showed moderate to stronger reduction (pPAB1 dropped 55%, pREV1 dropped 70%).

Due to the scarcity of functional data at this scale, the ANN system (*Place-Back* and *Determiner*) is trained on a self-supervised proxy objective of “contextual consistency”—specifically, recovering the Original Query Position Region (OQPR) from artificially corrupted sequences—rather than on functional repression data. Consequently, the model’s outputs represent contextual compatibility, not a direct prediction of TF binding specificity or regulatory performance. While the model effectively identifies insertion sites that accommodate the TFBS sequence without disrupting the “natural” syntax, it does not explicitly optimize for the maintenance of basal activity levels. The observed reduction in basal activity in pPAB1 and pREV1 likely reflects a trade-off where the most “naturally” fitting insertion site from the model's view structurally impacts the promoter’s native constitutive drive.

To verify that the model truly identifies specific functional “sweet spots” rather than relying on the inherent strength of the tetO operator, we analyzed two sets of negative controls. First, we tested the pFIG1 promoter, which the model classified as “incompatible”. As predicted, the recombinant pFIG1 construct was non-functional. Second, we compared the model’s recommended designs against alternative insertion sites within the same promoters that were not proposed by the model. These alternative placements frequently resulted in broken promoter logic; for instance, the alternative pPOP6 design exhibited 94% disruption of basal activity and failed to repress, whereas the model-guided design reduced disruption to 19% and achieved 98.4% repression (Supplementary Table 1). This contrast confirms that tetO insertion is not universally tolerated and that the model’s specific placement predictions are critical for function.

These results confirm that ANN-based design can yield strong, tunable repression and that the models can correctly identify sites with minimal disruption to native promoter activity. The orthogonal design enabled clean evaluation of promoter behavior in isolation.

### Rewiring of the yeast transcriptional network

To demonstrate that the system can be used not only for orthogonal control but also for rewiring native transcriptional regulation, we engineered the promoter of PCF11, an essential gene^41^, by inserting a Mig1 repressor binding site (mig1O: GTATTAAACCCGGGGTA^42^).

**Fig. 4:**
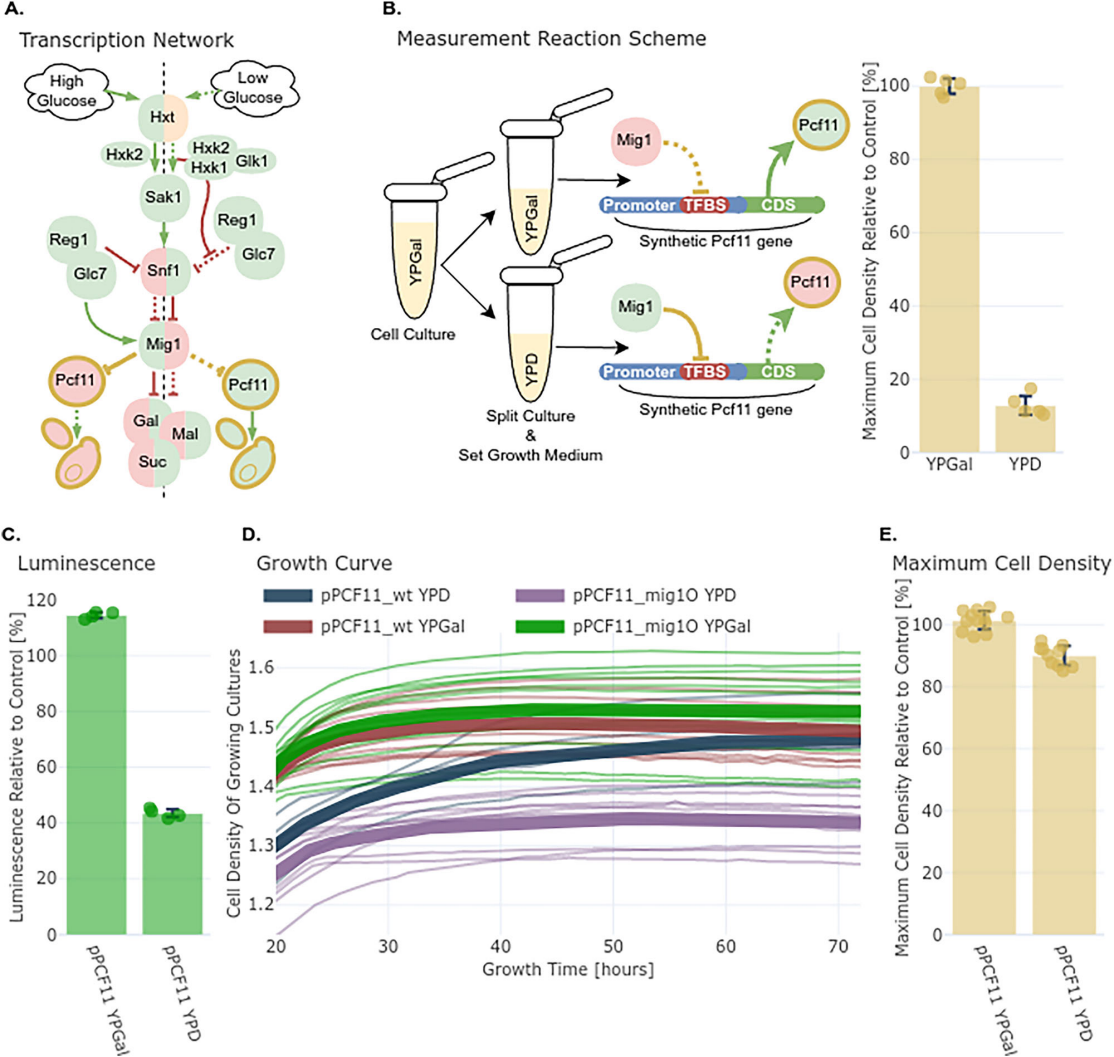
ANN-guided rewiring, insertion of Mig1 TFBS into the essential PCF11 promoter enables glucose-dependent transcriptional repression and growth control. **A** **Schematic**: The PCF11 promoter was modified with a Mig1 binding site (mig1O), creating a new regulatory edge in the glucose repression network. **B** **Experimental workflow**: Strains with synthetic and wild-type PCF11 promoters were tested in glucose (Mig1 ON) and galactose (Mig1 OFF). **C** **Luciferase assay**: In glucose, pPCF11_mig1O showed 57% repression compared to wild-type; in galactose, expression was slightly higher than wild-type. **D** **Growth curves**: Strains with the synthetic promoter showed 10% reduced maximum cell density in glucose and 1.4% increased in galactose, consistent with conditional repression of an essential gene. **E** **Growth summary**: Bar plot showing the relative change in maximal cell density. The results confirm successful network rewiring with regulatory effects that are strong but not lethal.

Mig1 is a glucose-sensitive transcriptional repressor active in high-glucose conditions (Fig. 4.A)^43–45^. Using the model, we inserted mig1O in the PCF11 promoter (Supplementary Fig. 17). Unlike the TetR system, this construct relied entirely on endogenous regulation, so no extra TFs were introduced.

First, we validated glucose-dependent regulation using NanoLuc assays. In galactose (Mig1 OFF, Fig. 4.B), the synthetic promoter showed 14% stronger activity than wild-type (Fig. 4.C). In glucose (Mig1 ON, Fig. 4.B), it was repressed by 57%.

Next, we replaced the endogenous PCF11 promoter in the yeast genome with the synthetic pPCF11_mig1O promoter using CRISPR-Cas9. Because PCF11 is essential, full repression would be expected to be lethal. Growth assays showed that in galactose, the strain with pPCF11_mig1O had slightly higher growth than wild-type (+1.4%), and in glucose, where pPCF11_mig1O is repressed by active Mig1, the strain had a 10% lower maximum cell density than wild-type, confirming repression but not full loss of function (Fig. 4.D, E).

This result is a key proof-of-concept: a single ANN-designed TFBS insertion created a new regulatory edge to rewire the yeast transcription network, controlling expression of an essential gene based on nutrient conditions, without additional synthetic TFs.

## Discussion

We developed a two-stage ANN system capable of guiding TFBS insertion into native promoter sequences in a context-aware manner. By combining self-supervised learning with promoter architecture analysis, our framework identifies viable insertion sites and proposes minimal, functional sequence modifications. The model was validated both in silico and in vivo, successfully generating synthetic promoters with strong repression in an orthogonal TetR system and enabling conditional control of an essential gene through rewiring of the native transcriptional network.

In the first phase, the implementation of an orthogonal repressor system using TetR-responsive promoters successfully introduced tunable transcriptional regulation into yeast. The designed synthetic promoters showed varying degrees of repression, with pPOP6_tetO achieving the highest repression rate of 98.4% and pPAB1_tetO reaching 90.1%. The analysis of promoter activity in wild-type versus engineered constructs indicated that while some promoters retained their original strength, others exhibited disruptions due to the introduction of TFBS. Despite these variations, the system effectively enabled external regulation of synthetic gene expression, demonstrating the power of ANN-driven promoter engineering.

The second phase of the study expanded upon these results by introducing a new regulatory edge within the native transcription network of *S. cerevisiae*. The pPCF11 promoter corresponding to the essential gene was engineered with a Mig1 repressor binding site, allowing for conditional repression based on glucose availability. Luciferase assays confirmed that the synthetic promoter responded to glucose-mediated repression, with a 57% repression rate in YPD medium. When the rewired regulatory system was implemented in the yeast genome, growth curve analysis revealed a 10% reduction in maximal cell density under high-glucose conditions, confirming that the introduced repression was functional but not lethal.

Overall, this study highlights the potential of ANN design in synthetic biology, enabling precise genetic modifications while minimizing unintended cellular disruptions. The success of both the orthogonal gene control and transcription network rewiring demonstrates that data-driven approaches can be leveraged for predictive and efficient genetic engineering.

Our current framework inserts the TFBS as a single contiguous block (e.g., the 40 bp tetO sequence). While previous studies suggest that separating operator sites can enhance repression efficiency through cooperative binding or nucleosome positioning^19^, we prioritized a single-query recombination approach to establish a baseline for model-guided insertion. However, we demonstrated that complex architectures can be achieved through iterative application of the model: a retrospective analysis using a two-step insertion strategy on promoters from^19^ showed that our model independently identified high-performing separated-site designs (matching the second-best experimental tetO construct (Supplementary Figs. 18–19), and matching the fourth-best experimental lacO construct (Supplementary Figs. 20–21)). Future iterations of the model could be trained to explicitly propose such split-site insertions to leverage these positional biases for tunable repression. Beyond these architectural refinements, we aim to extend the ANN models’ applicability from *S. cerevisiae* to a broader range of eukaryotic organisms, including plants, invertebrates, and vertebrates. Furthermore, the model and the generated dataset of context-aware insertions provide a foundation for future systematic studies to decode the implicit syntax of promoter architecture and derive new biological design principles.

## Methods

### Strains and plasmids preparation

All experiments were performed in *S. cerevisiae*. The yeast strain BY4741 was used as the parental strain (see Table 1 for full genotypes). To create a strain expressing the TetR repressor, a cassette containing pCCW12-TetR and a LEU2 selection marker was integrated into BY4741, resulting in strain S100. A control strain (S101) was constructed by integrating an empty LEU2 cassette.

**Table 1:** Strains

Strain	Genotype	Reference
BY4741	MATa his3∆1 leu2∆0 met15∆0 ura3∆0	^49^
S100	BY4741 leu2::pCCW12-TetR	
S101	BY4741 leu2::empty cassette	
S102	BY4741 leu2::pCCW12-TetR his3::pHHF2_wt-NanoLuc	
S103	BY4741 leu2::empty cassette his3::pHHF2_wt-NanoLuc	
S104	BY4741 leu2::pCCW12-TetR his3::pHHF2_tetO-NanoLuc	
S105	BY4741 leu2::empty cassette his3::pHHF2_tetO-NanoLuc	
S106	BY4741 leu2::pCCW12-TetR his3::pPAB1_wt-NanoLuc	
S107	BY4741 leu2::empty cassette his3::pPAB1_wt-NanoLuc	
S108	BY4741 leu2::pCCW12-TetR his3::pPAB1_tetO-NanoLuc	
S109	BY4741 leu2::empty cassette his3::pPAB1_tetO-NanoLuc	
S110	BY4741 leu2::pCCW12-TetR his3::pPOP6_wt-NanoLuc	
S111	BY4741 leu2::empty cassette his3::pPOP6_wt-NanoLuc	
S112	BY4741 leu2::pCCW12-TetR his3::pPOP6_tetO-NanoLuc	
S113	BY4741 leu2::empty cassette his3::pPOP6_tetO-NanoLuc	
S114	BY4741 leu2::pCCW12-TetR his3::pREV1_wt-NanoLuc	
S115	BY4741 leu2::empty cassette his3::pREV1_wt-NanoLuc	
S116	BY4741 leu2::pCCW12-TetR his3::pREV1_tetO-NanoLuc	
S117	BY4741 leu2::empty cassette his3::pREV1_tetO-NanoLuc	
S200	BY4741 his3::pPCF11_wt-NanoLuc	
S201	BY4741 his3::pPCF11_mig1O-NanoLuc	
S300	BY4741 pPCF11::pPCF11_mig1O	
S400	BY4741 leu2::pCCW12-TetR his3::pHHF2_tetO_alternative-NanoLuc	
S401	BY4741 leu2::empty cassette his3::pHHF2_tetO_alternative-NanoLuc	
S402	BY4741 leu2::pCCW12-TetR his3::pPAB1_tetO_alternative-NanoLuc	
S403	BY4741 leu2::empty cassette his3::pPAB1_tetO_alternative-NanoLuc	
S404	BY4741 leu2::pCCW12-TetR his3::pPOP6_tetO_alternative-NanoLuc	
S405	BY4741 leu2::empty cassette his3::pPOP6_tetO_alternative-NanoLuc	
S406	BY4741 leu2::pCCW12-TetR his3::pREV1_tetO_alternative-NanoLuc	
S407	BY4741 leu2::empty cassette his3::pREV1_tetO_alternative-NanoLuc	
S408	BY4741 leu2::pCCW12-TetR his3::pFIG1_wt-NanoLuc	
S409	BY4741 leu2::empty cassette his3::pFIG1_wt-NanoLuc	
S410	BY4741 leu2::pCCW12-TetR his3::pFIG1_tetO-NanoLuc	
S411	BY4741 leu2::empty cassette his3::pFIG1_tetO-NanoLuc	

Promoters selected for testing (pHHF2, pPAB1, pPOP6, pREV1) were obtained as domesticated part plasmids from the MoClo Yeast Toolkit^46^ and represented a range of constitutive expression strengths. These promoters are referred to as pX_wt, where “X” is the gene source, and “wt” indicates wild-type sequence.

### Construction of synthetic promoters with tetr binding sites

To insert TetR operator sequences (tetO) into native promoters, we used a scarless split-PCR method guided by the ANN model predictions. Each promoter was split at a model-predicted insertion point with a gap based on the selected MSRL of 5 bp or 40 bp into two fragments: pX_frag1 (upstream of insertion) and pX_frag2 (downstream of insertion). Restriction sites were strategically placed to allow seamless assembly of the fragments flanking the inserted tetO sequence, avoiding any extraneous nucleotides at junctions.

- pX_frag1 included a 5′ overhang with the upstream portion of tetO and a BsmBI site for joining with pX_frag2, and a 3′ BsaI/BsmBI site for cloning.
- pX_frag2 contained the complementary BsmBI site and the downstream portion of tetO in its 3′ overhang, and a 5′ BsaI/BsmBI site

The two promoter fragments with tetO insert were assembled via BsmBI Golden Gate assembly into a CamR-marked bacterial entry vector (PEV), producing synthetic promoter constructs referred to as pX_tetO, where “tetO” refers to promoter version recombined with TFBS tetO. Assembly products were transformed into *Escherichia coli* NEB Turbo cells and screened via colony PCR and sequencing.

### Assembly of expression constructs

Final expression cassettes were assembled using BsaI Golden Gate reactions. Each construct contained:

- A synthetic or wild-type promoter (pX_tetO or pX_wt),
- His3 selection marker (with AmpR for *E. coli* propagation),
- MFW PrePro signal peptide,
- NanoLuc reporter^47^,
- tENO2 terminator.

Following transformation into NEB Turbo *E. coli*, plasmid miniprep was performed, and constructs were validated by BsmBI restriction digestion and gel electrophoresis.

### Genomic integration into *Saccharomyces cerevisiae*

Final gene constructs were integrated into *S. cerevisiae* strains S100 and S101 at designated orthogonal loci via yeast transformation and plated on His3 selection media, resulting in strains S102-S117. Successful genomic integration was confirmed through NotI digestion analysis and colony PCR.

### Luciferase assays for promoter rewiring experiments

To assess transcriptional rewiring, the native pPCF11 promoter (750 bp upstream of the ORF) was amplified and modified by insertion of a Mig1 operator sequence (mig1O), using the same scarless split-PCR strategy described above. The resulting synthetic promoter (pPCF11_mig1O) and wild-type version (pPCF11_wt) were assembled with NanoLuc, HIS3, and tENO2, then integrated into BY4741, producing strains S200 and S201 for luciferase-based expression analysis.

### CRISPR-Cas9 mediated genomic rewiring

Precise genomic integration of pPCF11_mig1O was performed using CRISPR-Cas9. A guide RNA targeting a PAM-adjacent site in the native pPCF11 promoter was cloned into a Cas9 expression plasmid with a *URA3* marker. The synthetic pPCF11_mig1O construct was supplied as a donor repair template. Co-transformation into BY4741 yielded strain S300. Transformants were selected on *URA3*-deficient media, and the Cas9 plasmid was subsequently removed via 5-FOA counter-selection. Correct integration was confirmed by PCR and sequencing.

### Gene expression analysis using luciferase assays

Promoter activity was measured using NanoLuc luciferase assays under varying environmental conditions.

#### TetO promoters

Cultures were grown in YPD media^48^, diluted to $O{D}_{600}=0.05$, and treated with 2 μg/mL doxycycline (DOX) to induce the ON state or left untreated for the OFF state. Relative Light Unit (RLU) was measured after 2 h in a white 96-well plate.

To ensure clarity, we defined $${RL}{U}_{{promoterType}}^{\pm {DOX}|\pm {TetR}}$$ as the luminescence of a specific strain with synthetic ($${syn}$$) or wild-type ($${wt})$$ promoter of interest, in the presence ($$+$$) or absence ($$-$$) of doxycycline ($${DOX}$$), in a strain constitutively expressing ($$+$$) or lacking ($$-$$) TetR repressor ($${TetR}$$). Metrics were calculated as follows:

First, to account for the metabolic burden or physiological effects of doxycycline on strains lacking the repressor, we calculated a **Correction factor** ($\delta$):

1 $$\delta =\frac{{RL}{U}_{{syn}}^{-{DOX|}-{TetR}}}{{RL}{U}_{{syn}}^{+{DOX|}-{TetR}}}$$

**Fold Induction** was calculated as the ratio of induced to uninduced signal, normalized by the correction factor:

2 $${Fold}\,{Induction}=\frac{{RL}{U}_{{syn}}^{+{DOX|}+{TetR}}}{{RL}{U}_{{syn}}^{-{DOX|}+{TetR}}}\times \delta$$

**Repression Rate** denotes the efficiency of the OFF state relative to the ON state, normalized by the correction factor:

3 $${Repression}\,{Rate}=1-\left(\frac{{RL}{U}_{{syn}}^{-{DOX|}+{TetR}}}{{RL}{U}_{{syn}}^{+{DOX|}+{TetR}}}\,/\,\delta \right)$$

**Disruption Rate** quantifies the loss of basal repression compared to the wild-type for strains lacking TetR:

4 $${Disruption}\,{Rate}=1-\frac{{RL}{U}_{{syn}}^{-{DOX|}-{TetR}}}{{RL}{U}_{{wt}}^{-{DOX|}-{TetR}}}$$

#### Mig1O promoters

Strains were cultured in YPGal media (YPD^48^ with glucose replaced by galactose). Cultures were diluted to $O{D}_{600}=0.05$ and split into YPD (high glucose, repression ON) and YPGal (low glucose, repression OFF). **Relative promoter activity** was determined as:

5 $${Relative}\,{Activity}=\frac{{RL}{U}_{{syn}}}{{RL}{U}_{{wt}}}$$

### Maximum cell density measurements for rewired network

To evaluate the effect of the rewired network (pPCF11_mig1O) on growth, strains BY4741 (wild-type pPCF11_wt) and S300 (synthetic pPCF11_mig1O) were cultured in YPGal, then washed and transferred to YPD or YPGal media. Cultures were diluted to $O{D}_{600}=0.05$ and monitored every hour for 72 h using a plate reader. Maximum cell density was calculated as:

6 $${Relative}\,{Max}\,{Cell}\,{Density}=\frac{{MaxO}{D}_{{syn}}}{{MaxO}{D}_{{wt}}}$$

### Statistical analysis

All experiments were performed in three biological replicates and two technical replicates in at least three independent days. Statistical significance was assessed using ANOVA, with a significance threshold of *p* < 0.05.

## Supplementary information

Supplementary Information

## Data Availability

The DNA sequences for all primary and control synthetic promoters have been deposited in the GenBank database under accession numbers PX984214–PX984223. The minimal dataset required to interpret and replicate the findings, including raw luciferase assay measurements and growth curve data, has been deposited in the Figshare repository under 10.6084/m9.figshare.31298335.

## Code availability

The promoter design software developed in this study is released under the GPL-3.0 license and can be accessed via GitHub at https://github.com/GeorgievLab/design_of_promoter_logic. A browser-based implementation is also available at https://www.kky.zcu.cz/en/Tools/placeback.
