# Supplementary material for: Context-aware synthetic promoter design using neural networks enables rewiring of eukaryotic transcriptional networks
Source: NPJ Syst Biol Appl. 2026 Mar 17;12:65. doi: 10.1038/s41540-026-00684-5 (PMC13144678; doi:10.1038/s41540-026-00684-5)
Supplement: Supplementary file 1 — Supplementary Information [file 41540_2026_684_MOESM1_ESM.docx]

**Supplementary Information**

**Data**

Recommended promoters for recombination with tetO: <https://github.com/GeorgievLab/design_of_promoter_logic/blob/main/data/promoters/promoters_for_tetO_recombination.json>

**Figures**


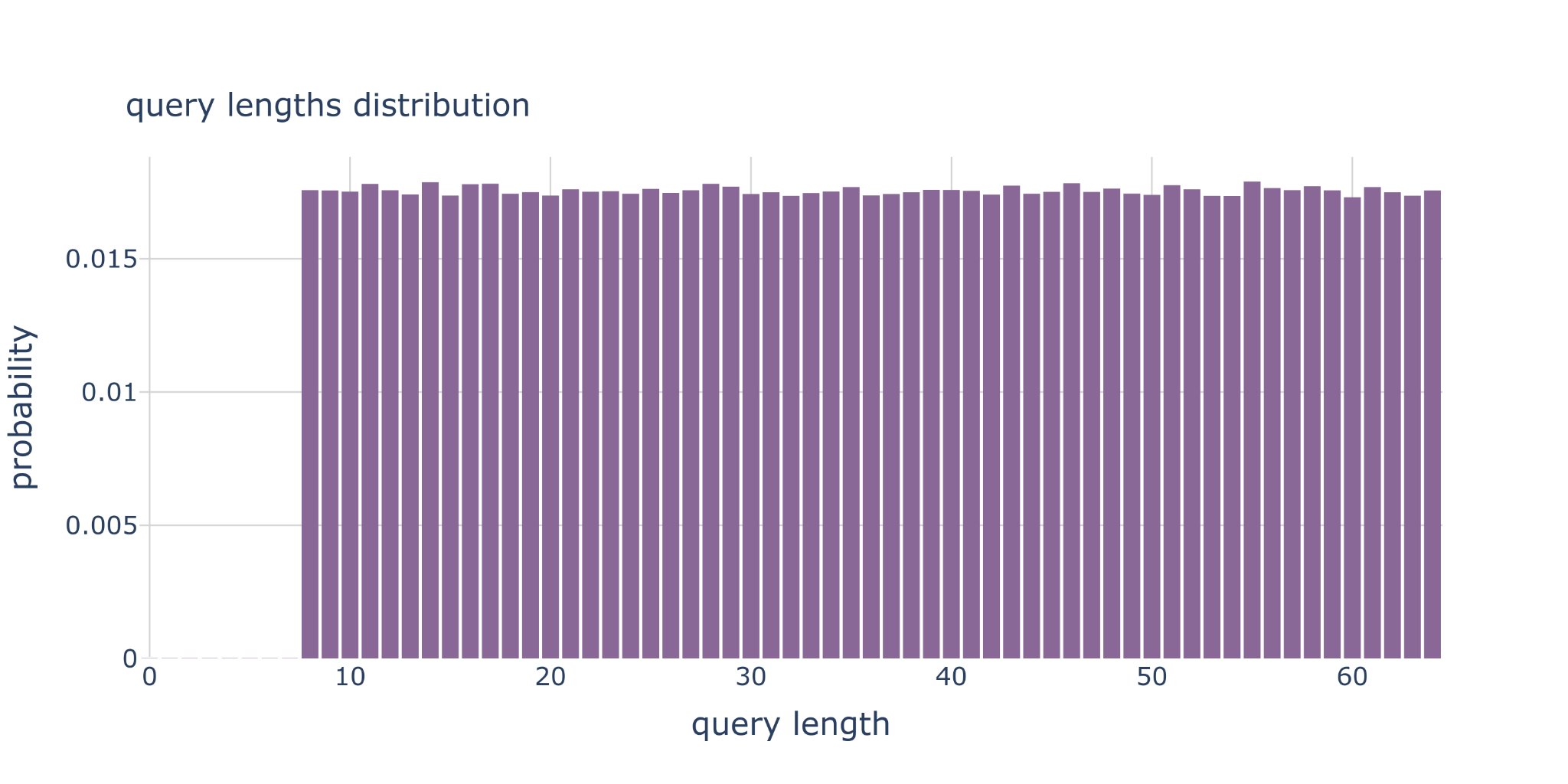


**Supplementary Figure 1**: Distribution of sampled query lengths used during the *Place-Back* model training process.


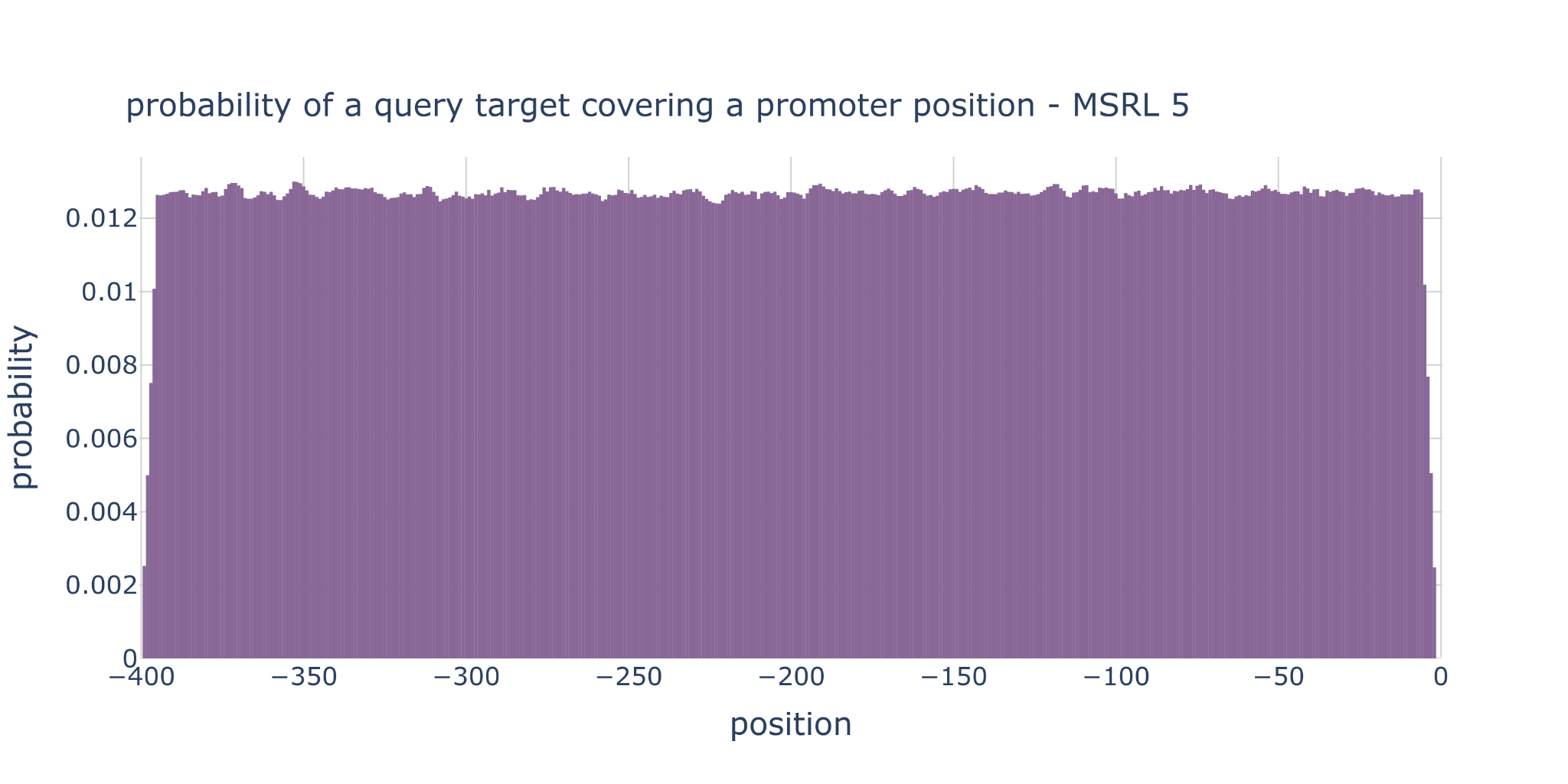


**Supplementary Figure 2**: Probability of a query target covering a specific promoter position within the inputs generated for training *Place-Back* models with a MSRL of 5 bp.


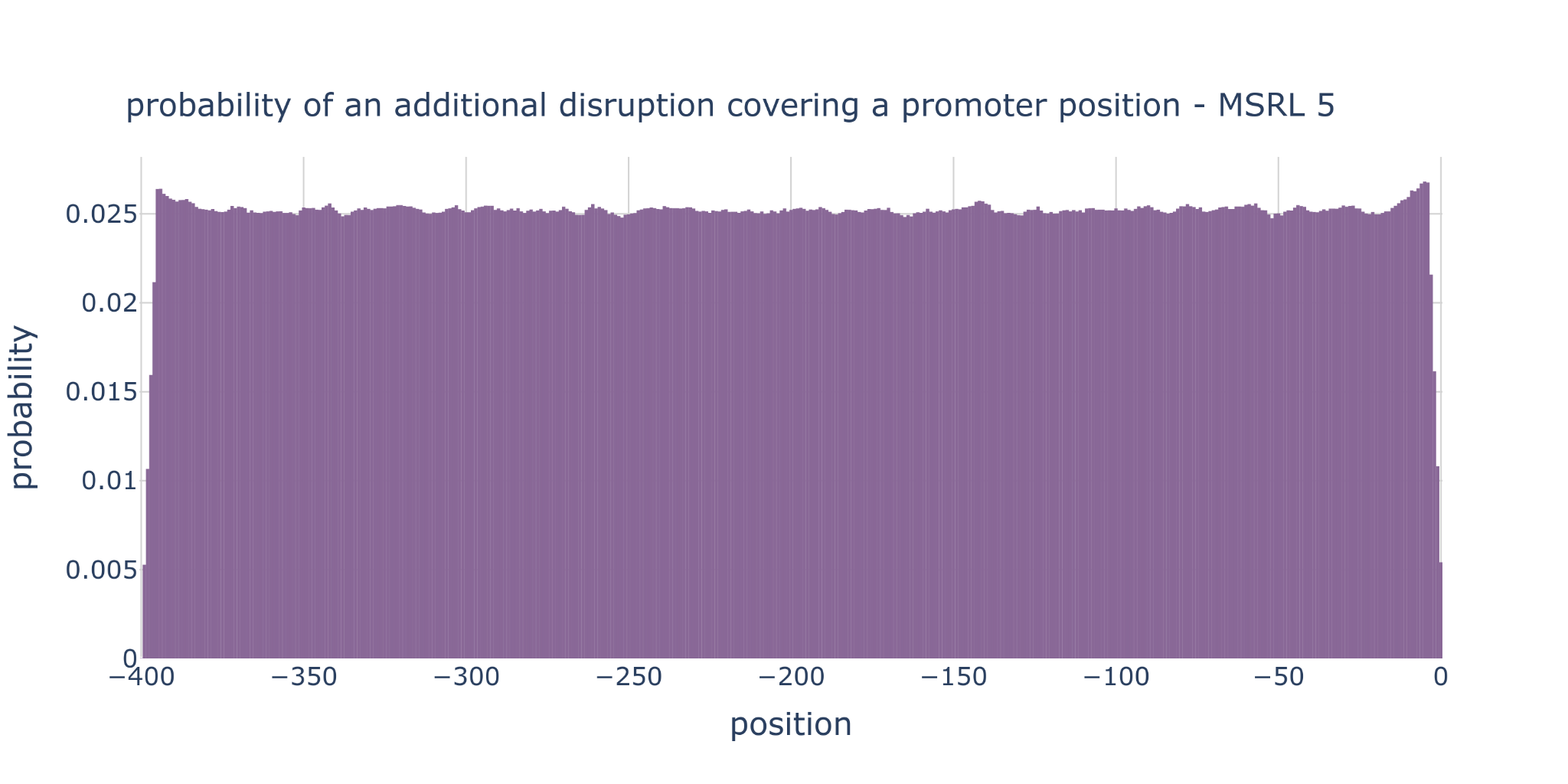


**Supplementary Figure 3**: Probability of an additional promoter disruption covering a specific promoter position within the inputs generated for training *Place-Back* models with a MSRL of 5 bp.


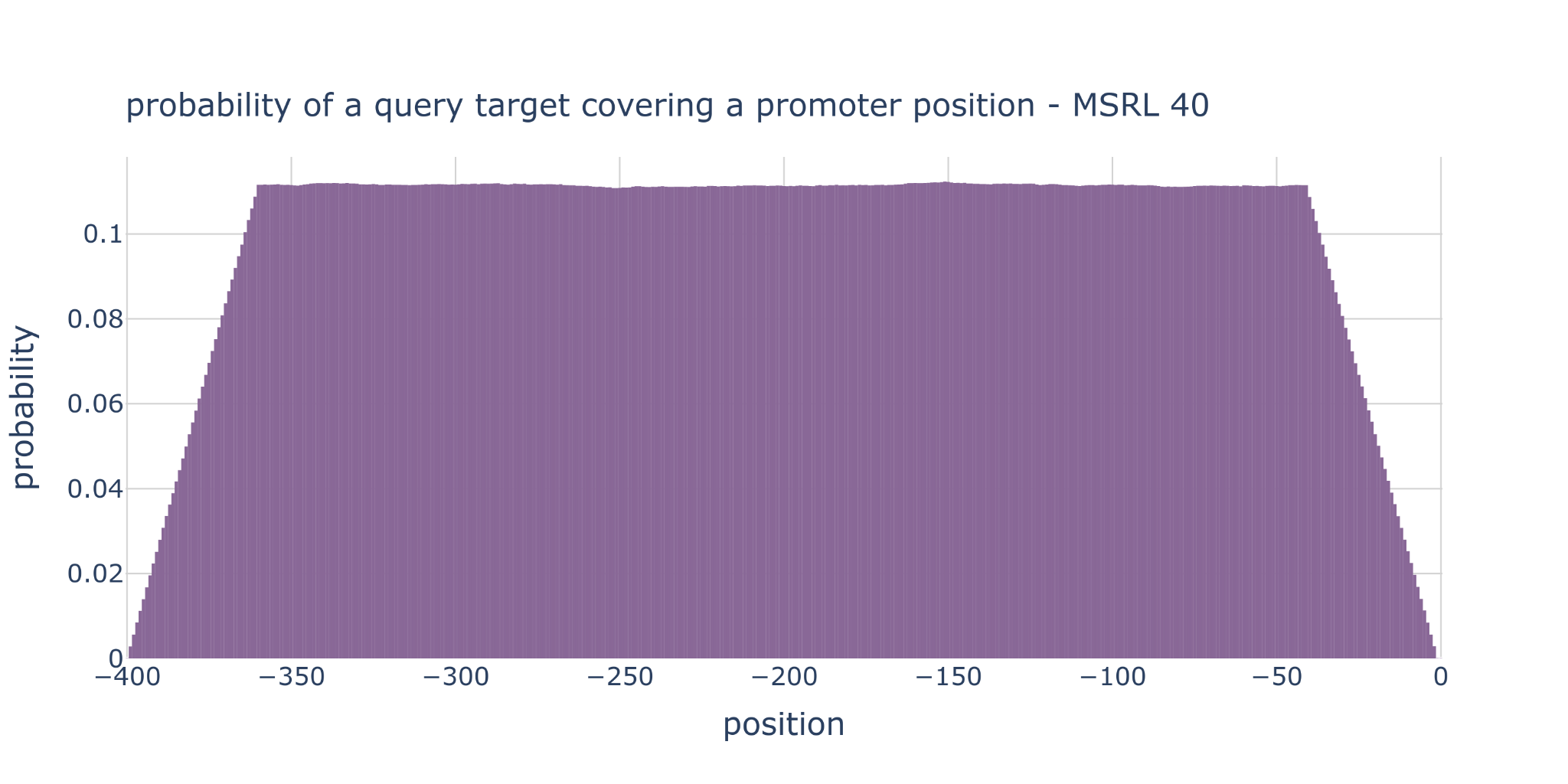


**Supplementary Figure 4**: Probability of a query target covering a specific promoter position within the inputs generated for training *Place-Back* models with a MSRL of 40 bp.


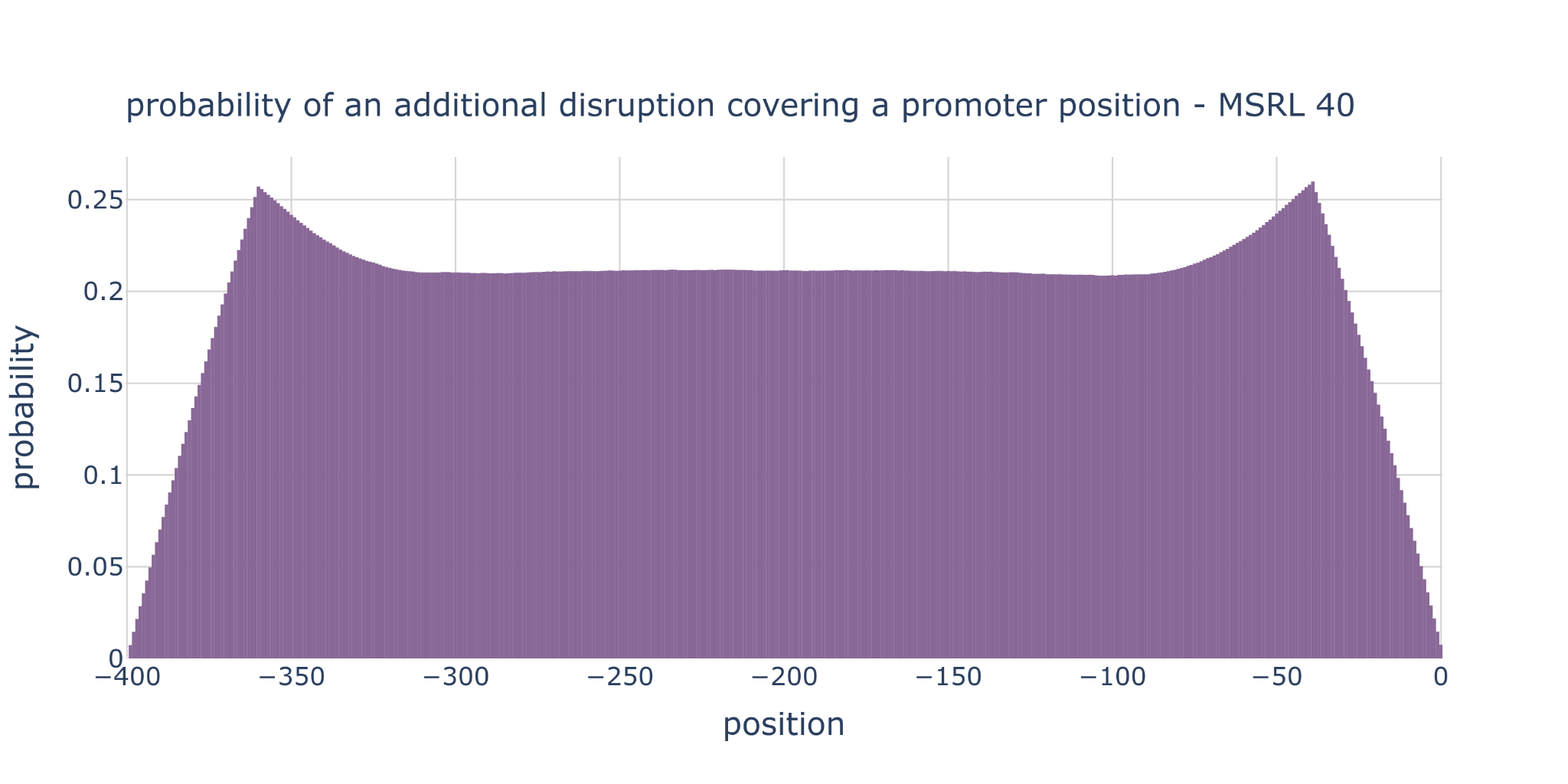


**Supplementary Figure 5**: Probability of an additional promoter disruption covering a specific promoter position within the inputs generated for training *Place-Back* models with a MSRL of 40 bp.


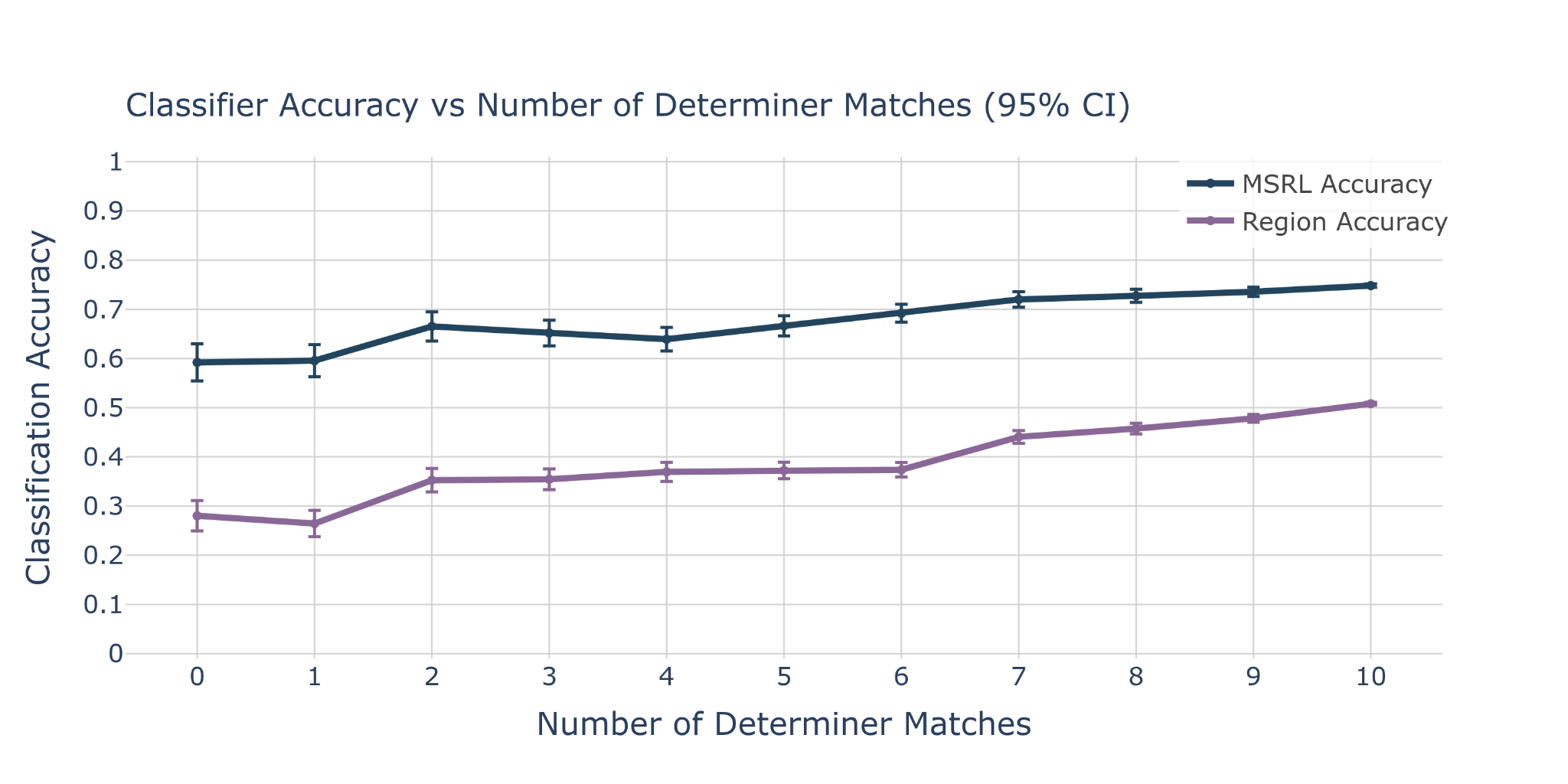


**Supplementary Figure 6**: Classification accuracy of the averaged *Determiner* model ensemble as a function of the number of models with internal consistency ("Match"). The highest accuracy is observed when all *Determiner* models produce a “Match” output. Generally, high-confidence accuracy is achieved when at least 7 out of 10 *Determiner* models yield a “Match”.


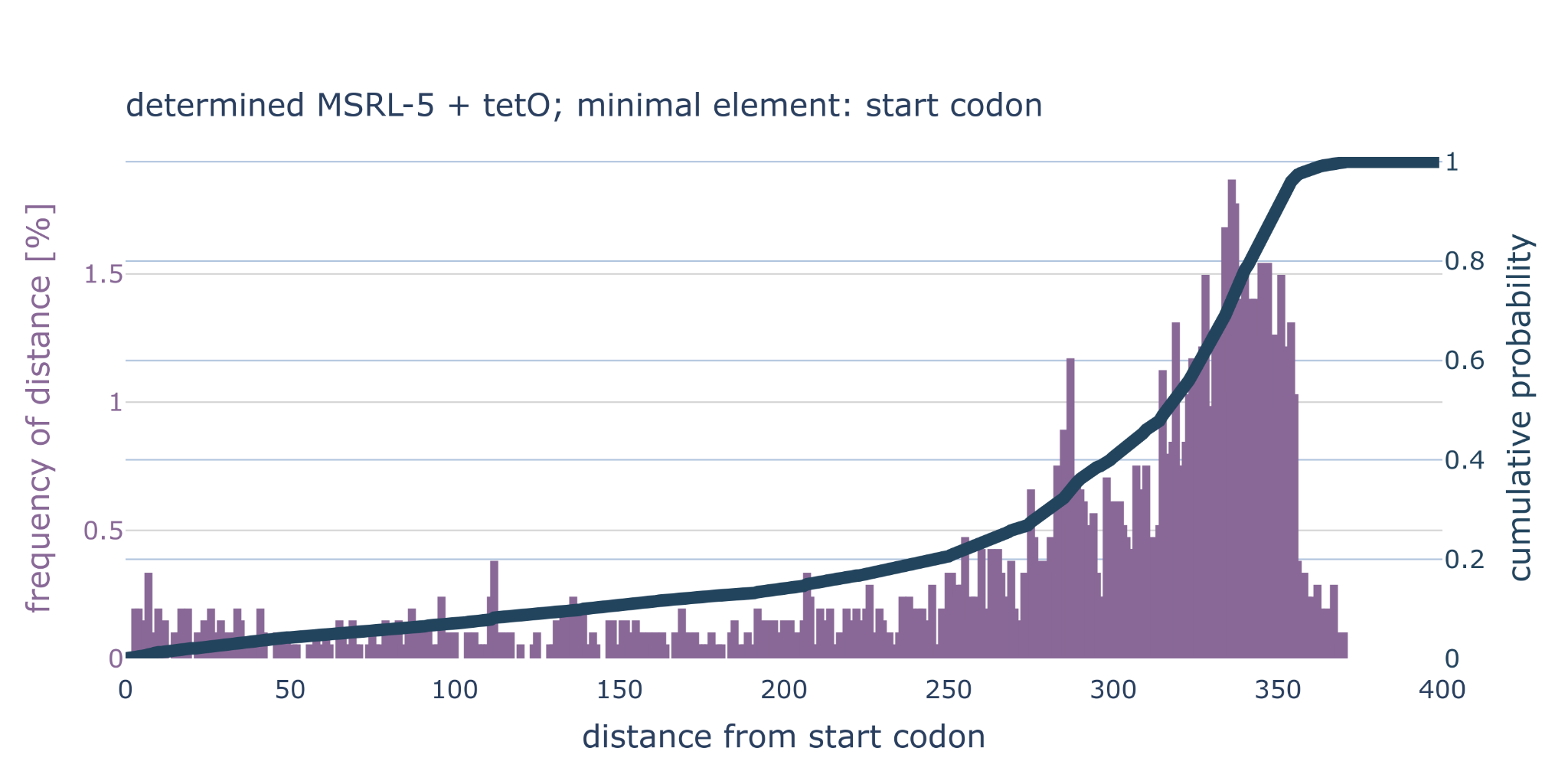


**Supplementary Figure 7**: Distribution of tetO placement distances relative to the start codon in recommended promoters when models with a MSRL of 5bp were selected.


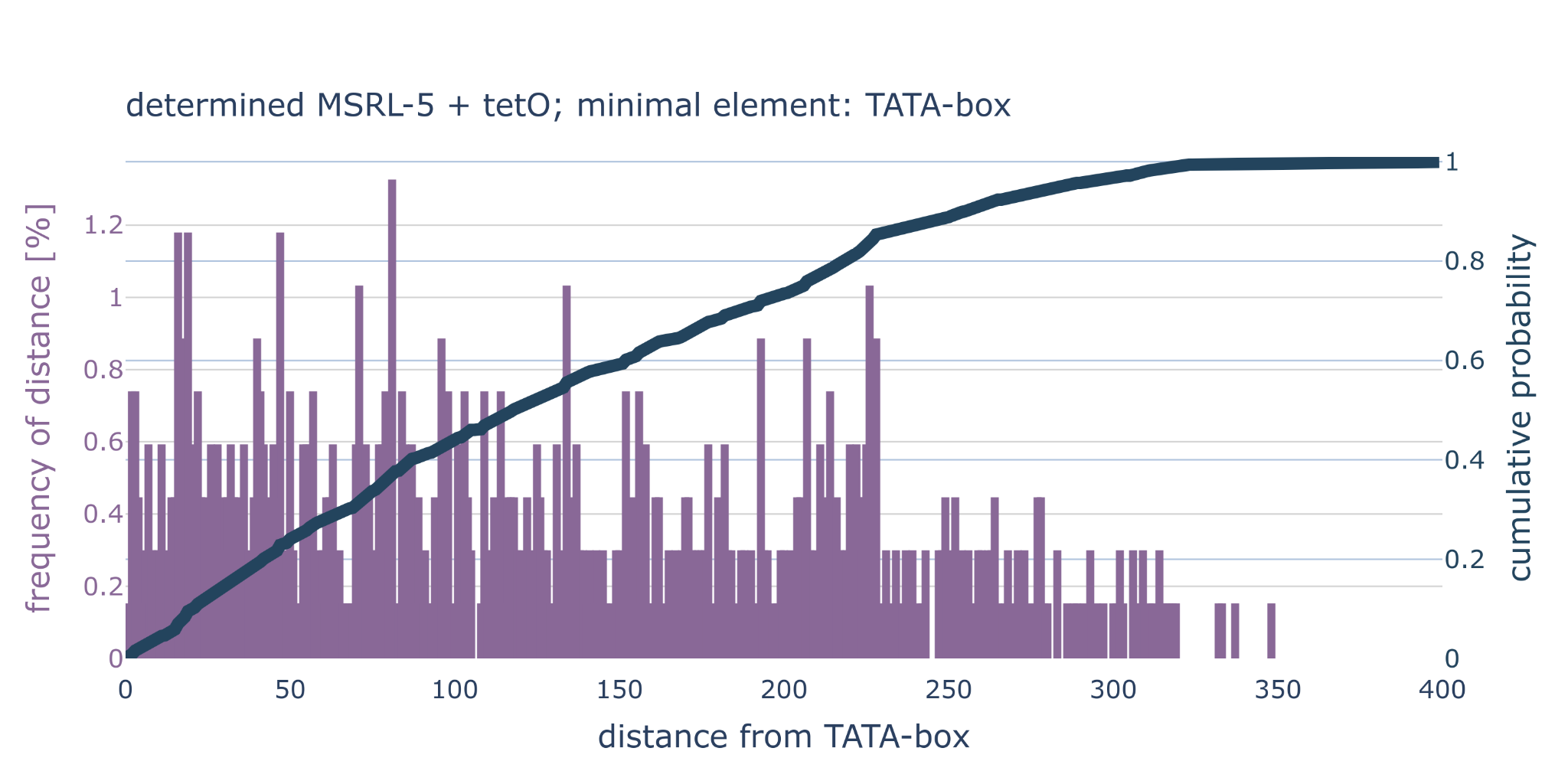


**Supplementary Figure 8**: Distribution of tetO placement distances relative to the TATA-box in recommended promoters when models with a MSRL of 5bp were selected.


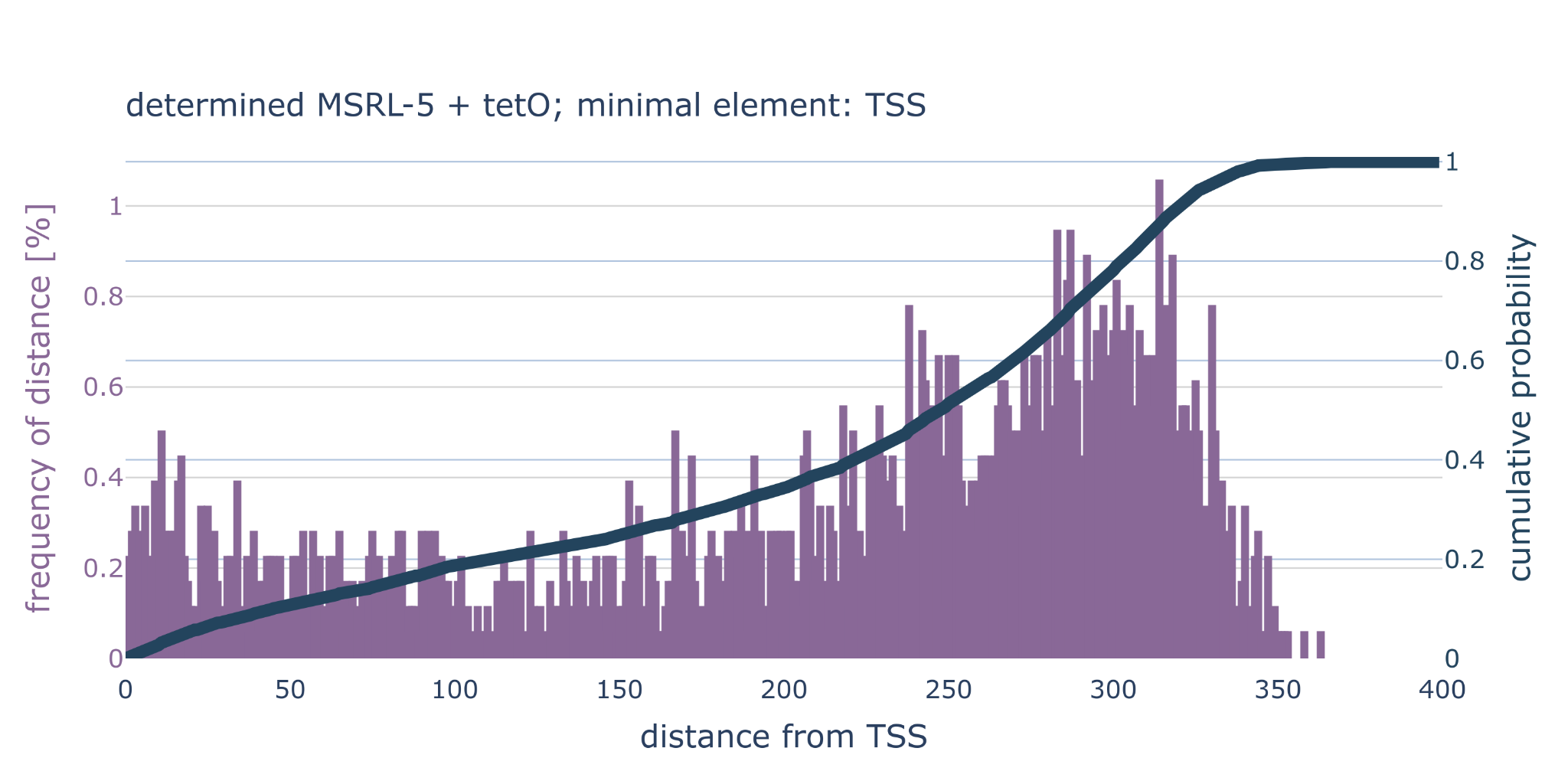


**Supplementary Figure 9**: Distribution of tetO placement distances relative to the TSS in recommended promoters when models with a MSRL of 5bp were selected.


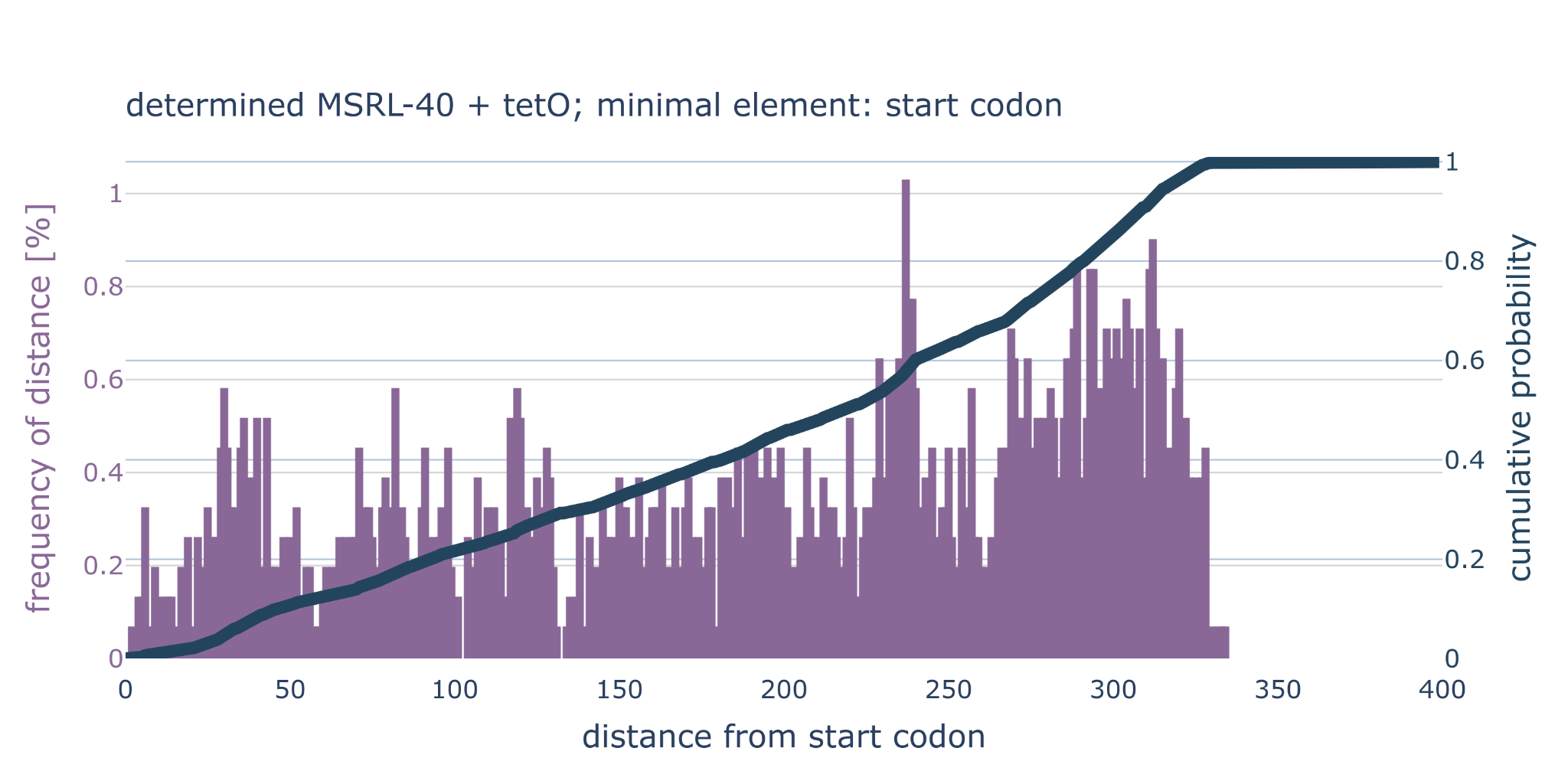


**Supplementary Figure 10**: Distribution of tetO placement distances relative to the start codon in recommended promoters when models with a MSRL of 40bp were selected.


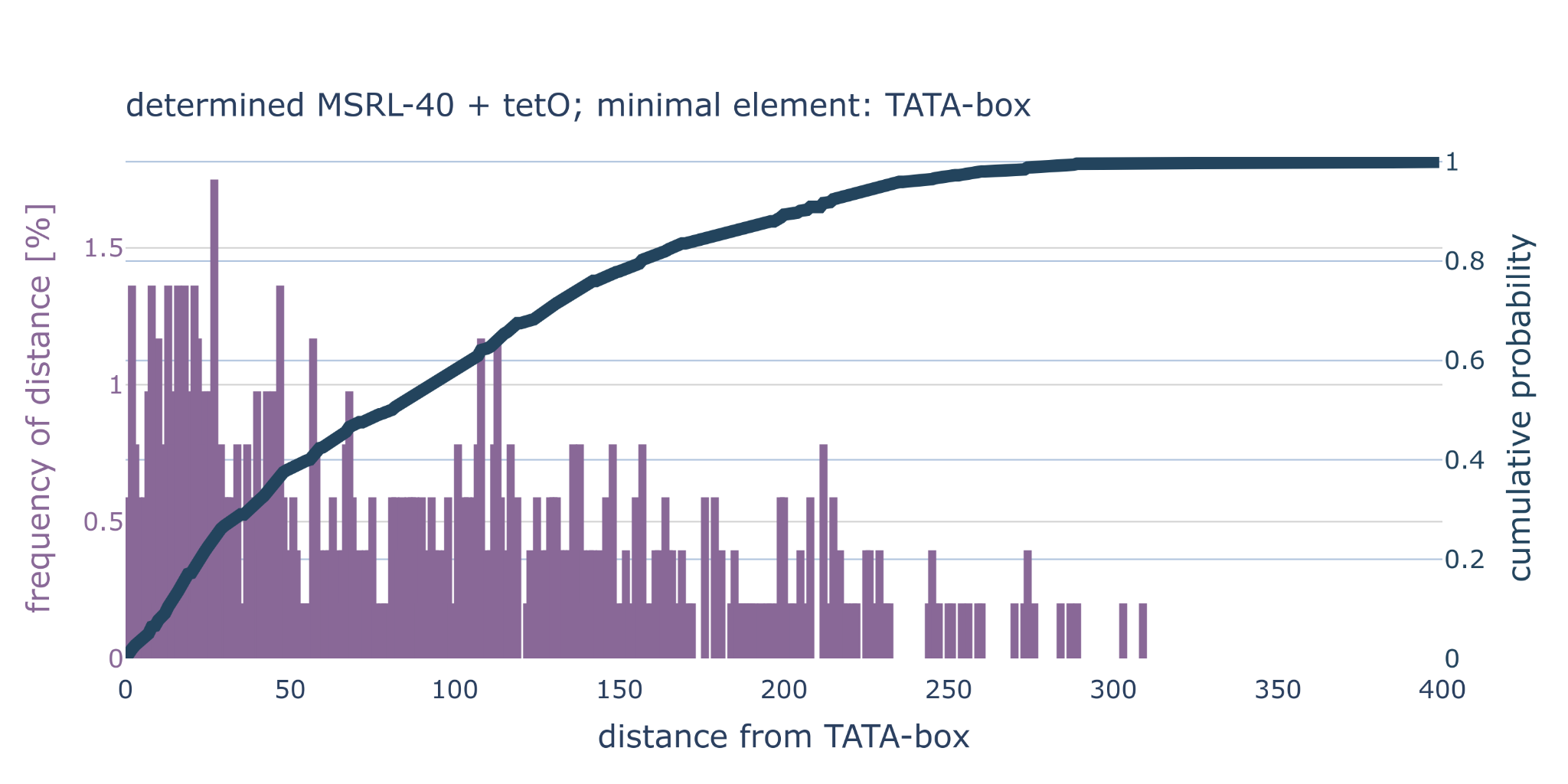


**Supplementary Figure 11**: Distribution of tetO placement distances relative to the TATA-box in recommended promoters when models with a MSRL of 40bp were selected.


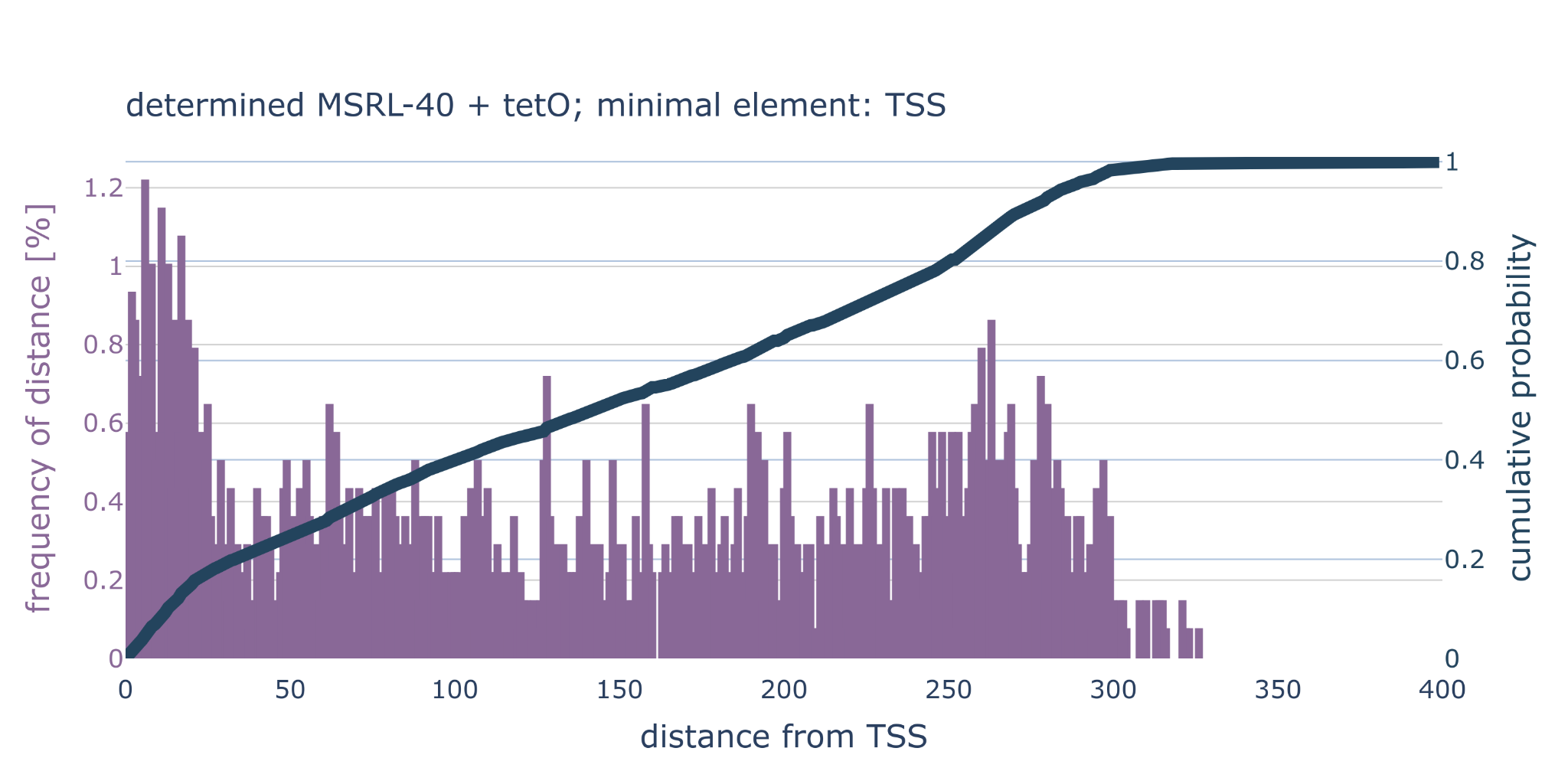
**Supplementary Figure 12**: Distribution of tetO placement distances relative to the TSS in recommended promoters when models with a MSRL of 40bp were selected.


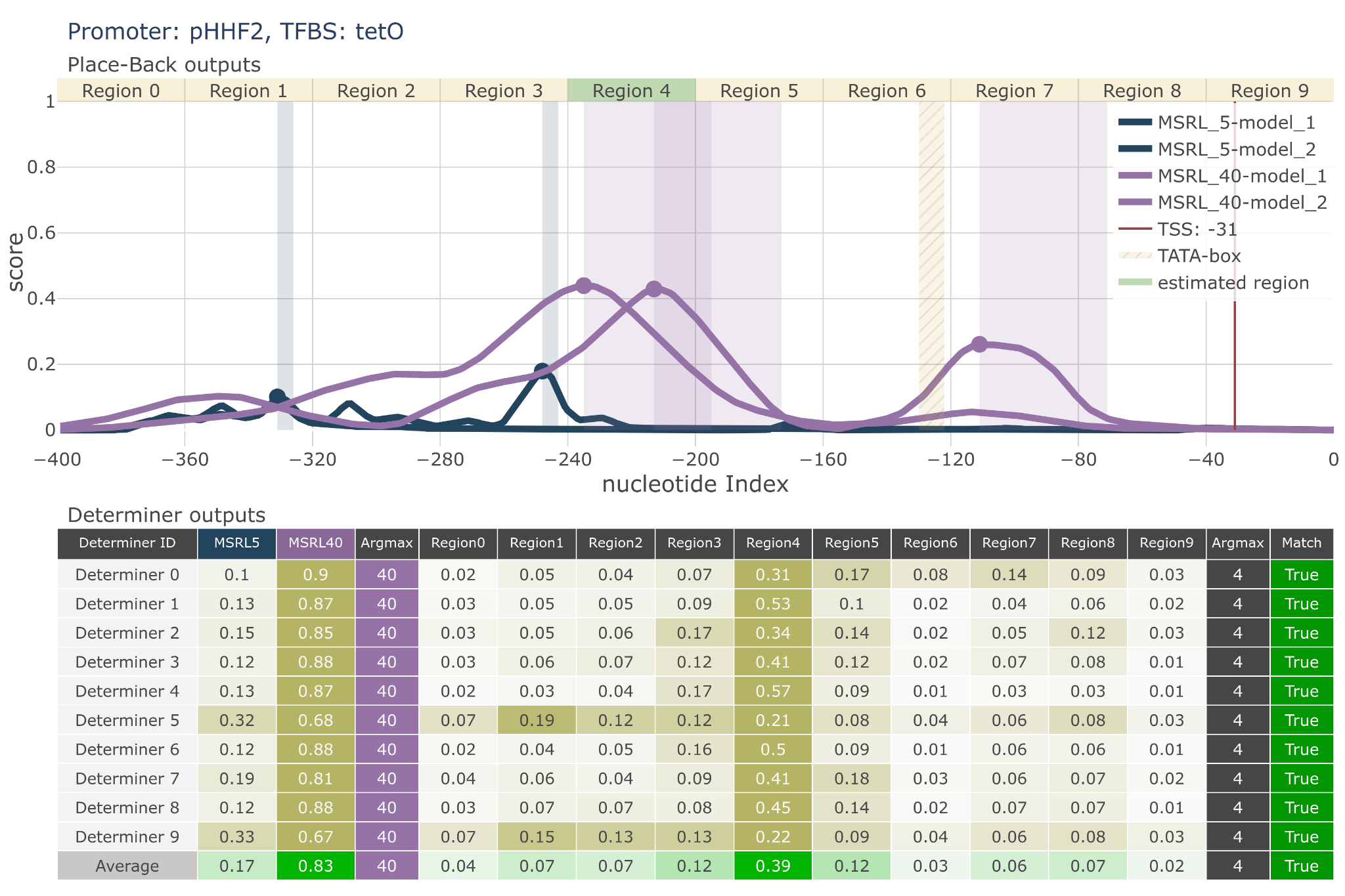


**Supplementary Figure 13**: Complete output from the two-stage ANN system for the recombination of the wild-type pHHF2 promoter with tetO. The region selected for experimental validation spans from -235 to -195 relative to the start codon.


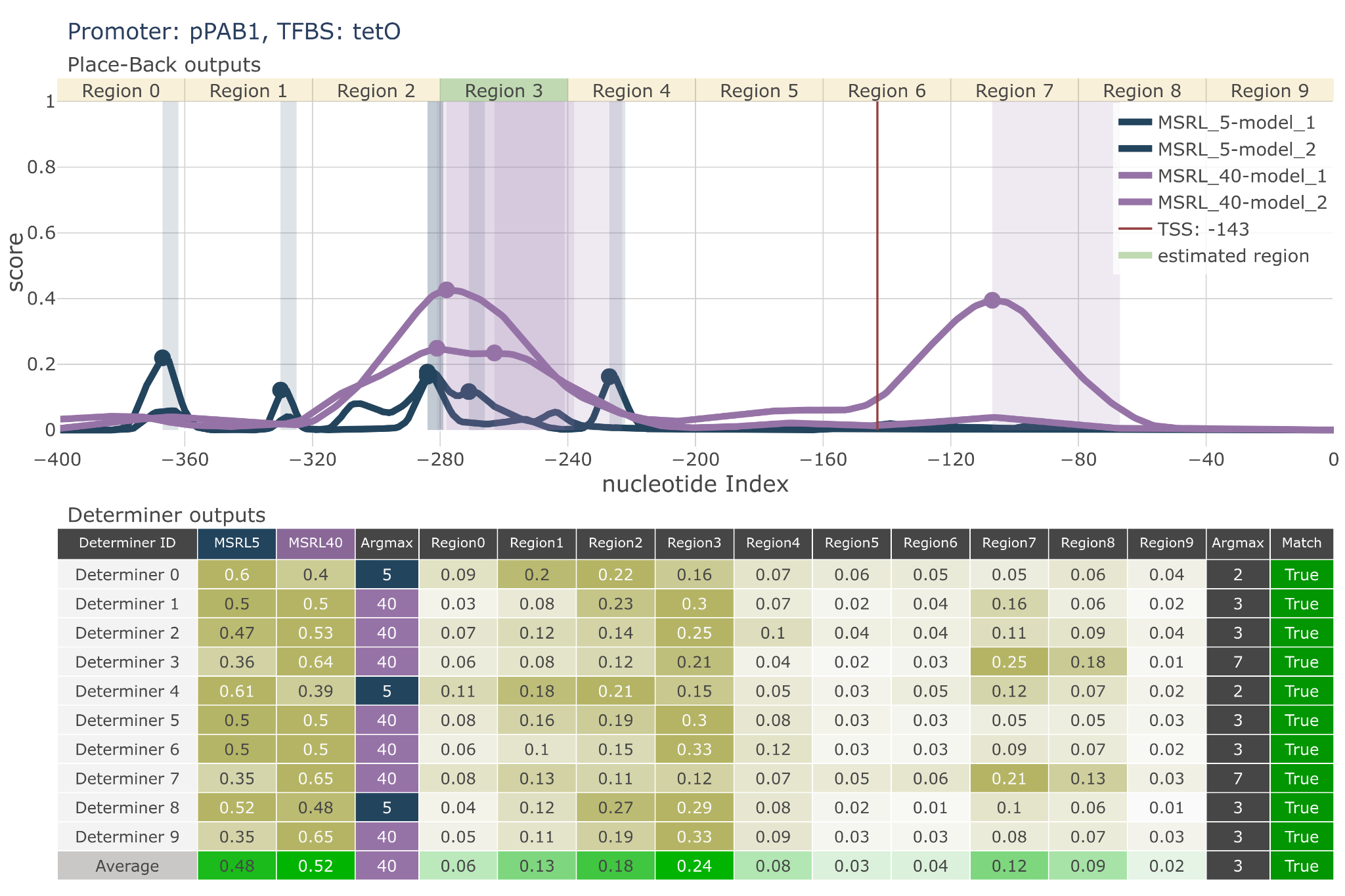


**Supplementary Figure 14**: Complete output from the two-stage ANN system for the recombination of the wild-type pPAB1 promoter with tetO. The region selected for experimental validation spans from -278 to -238 relative to the start codon.


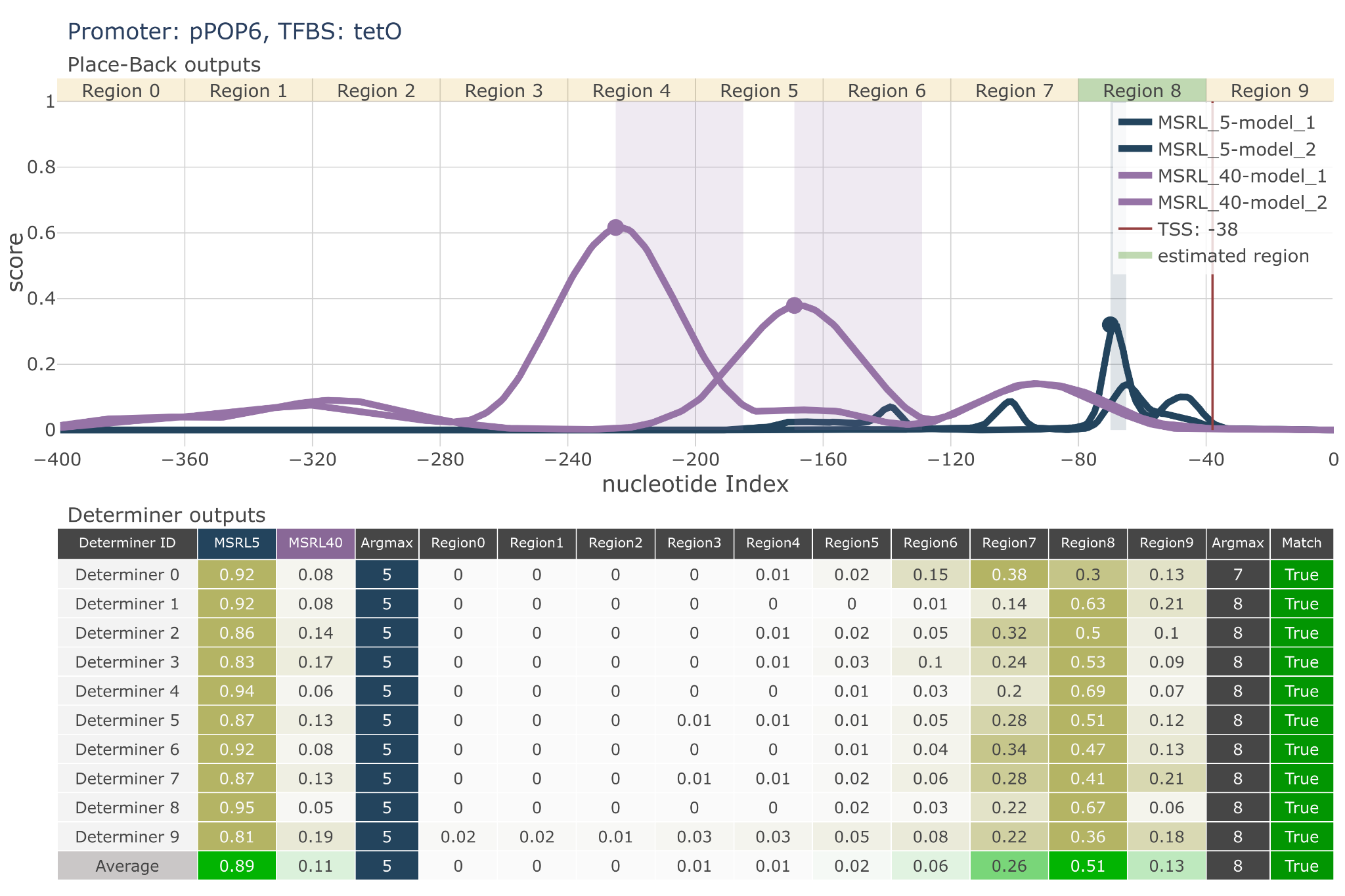


**Supplementary Figure 15**: Complete output from the two-stage ANN system for the recombination of the wild-type pPOP6 promoter with tetO. The region selected for experimental validation spans from -70 to -65 relative to the start codon.


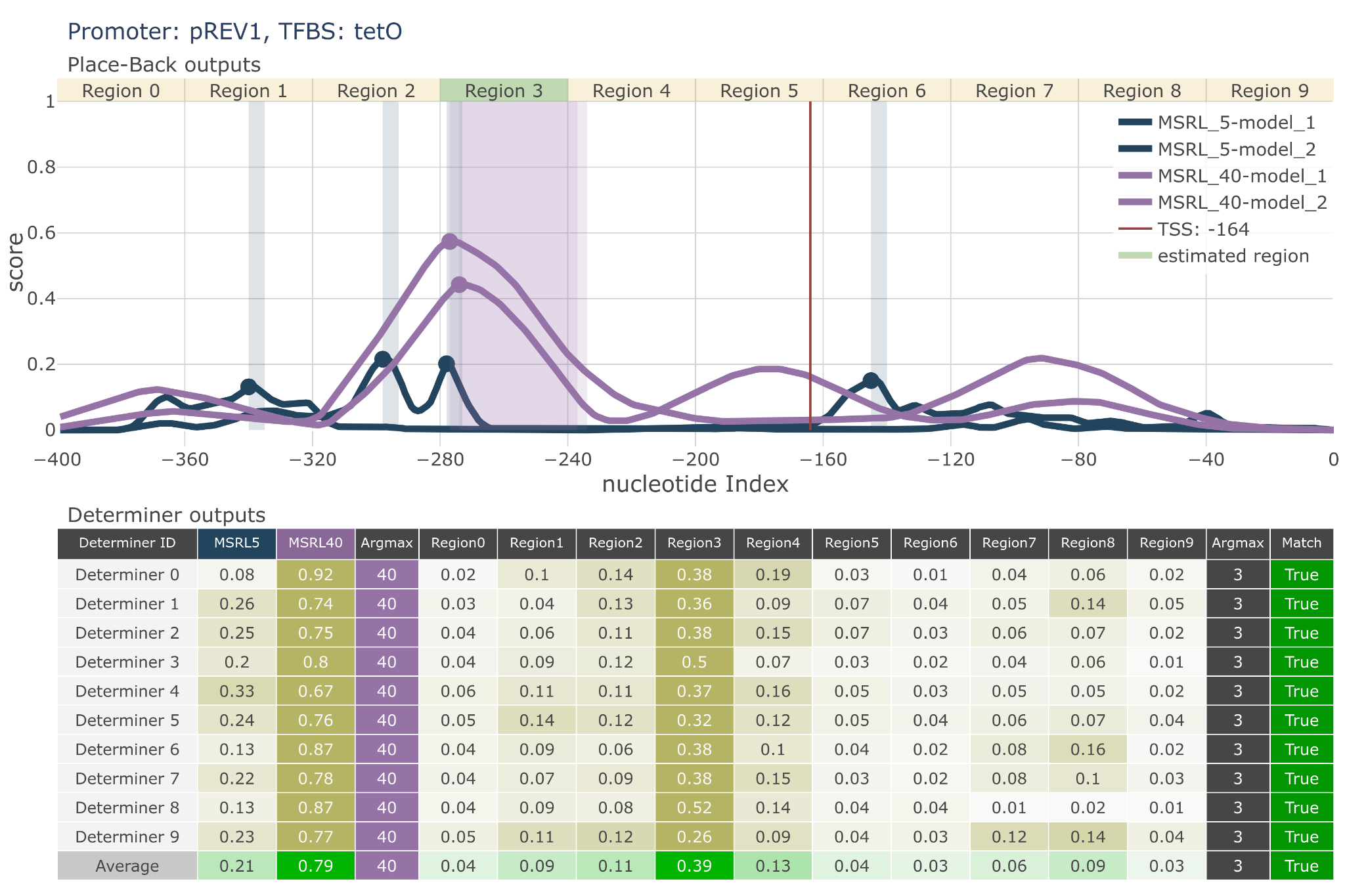


**Supplementary Figure 16**: Complete output from the two-stage ANN system for the recombination of the wild-type pREV1 promoter with tetO. The region selected for experimental validation spans from -277 to -237 relative to the start codon.


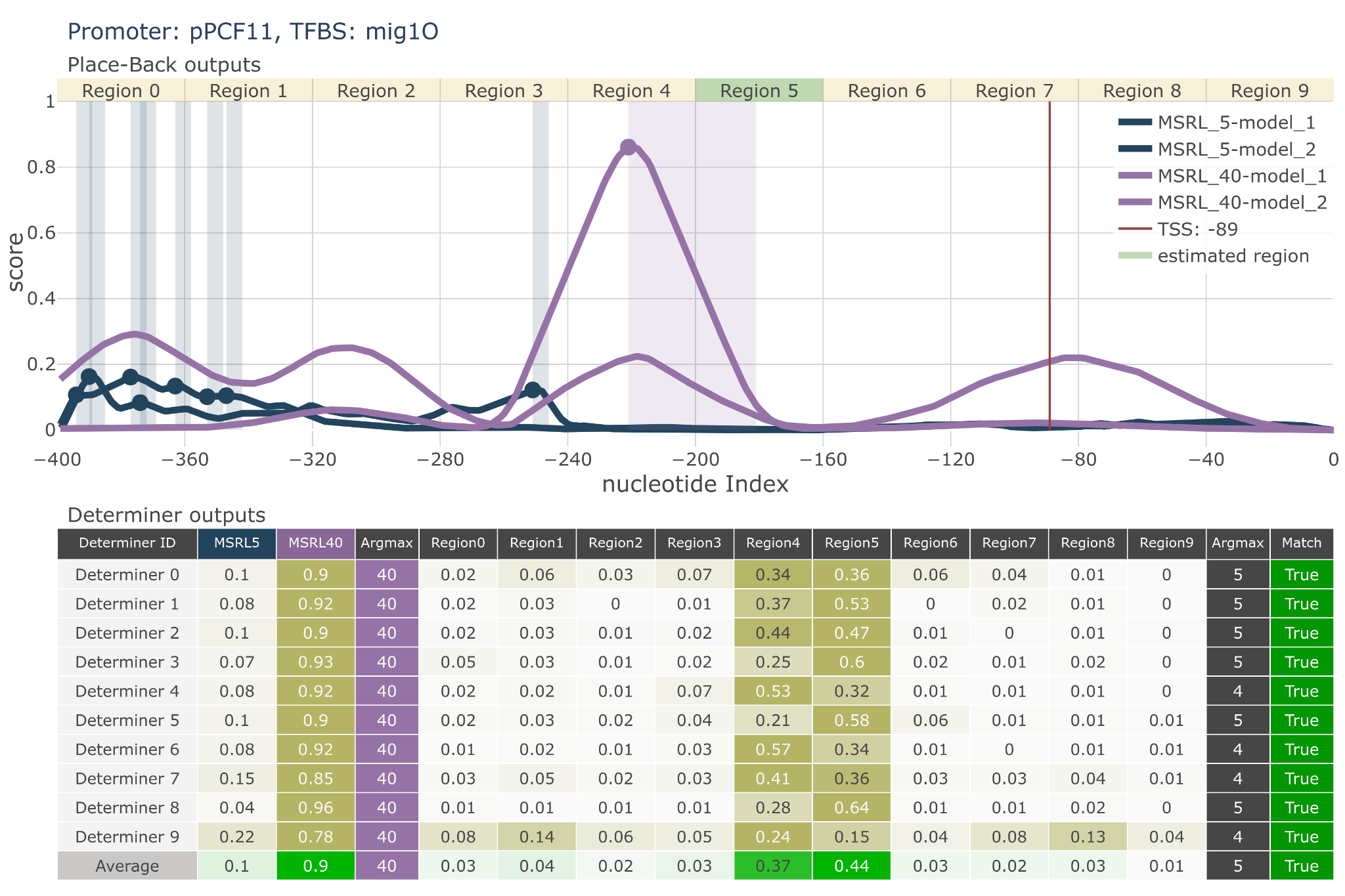


**Supplementary Figure 17**: Complete output from the two-stage ANN system for the recombination of the wild-type pPCF11 promoter with mig1O. The region selected for experimental validation spans from -221 to -181 relative to the start codon.


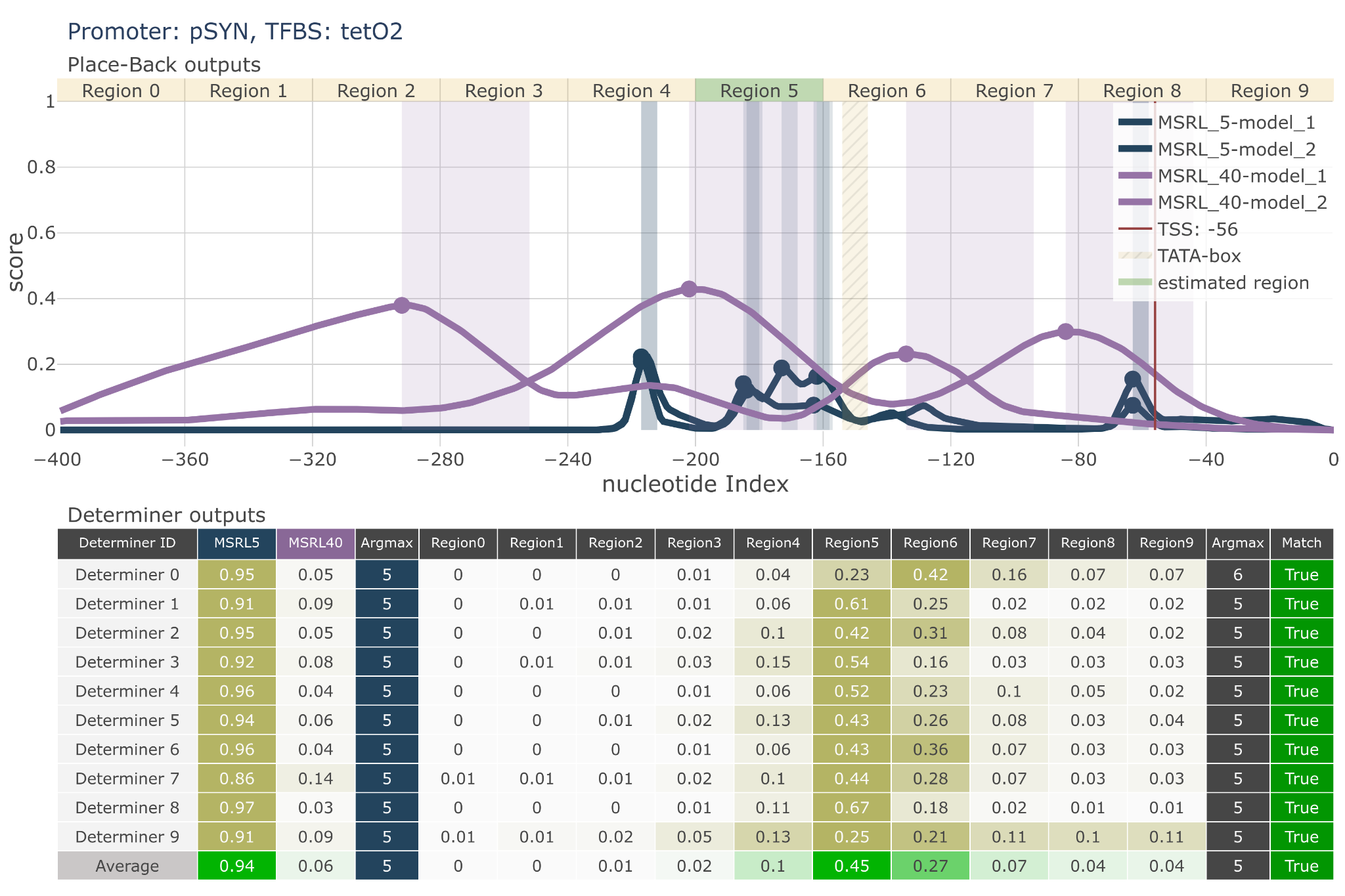


**Supplementary Figure 18**: Complete output from the two-stage ANN system for recombination of the pSYN promoter [19] with tetO2 [19]. The selected tetO2 placement is from -173 to -168, mimicking Psyn.tetO2D construct from [19]. The first-round modification (single-site tetO2) serves as the input promoter for the second round of design (see Supplementary FIg. 19).


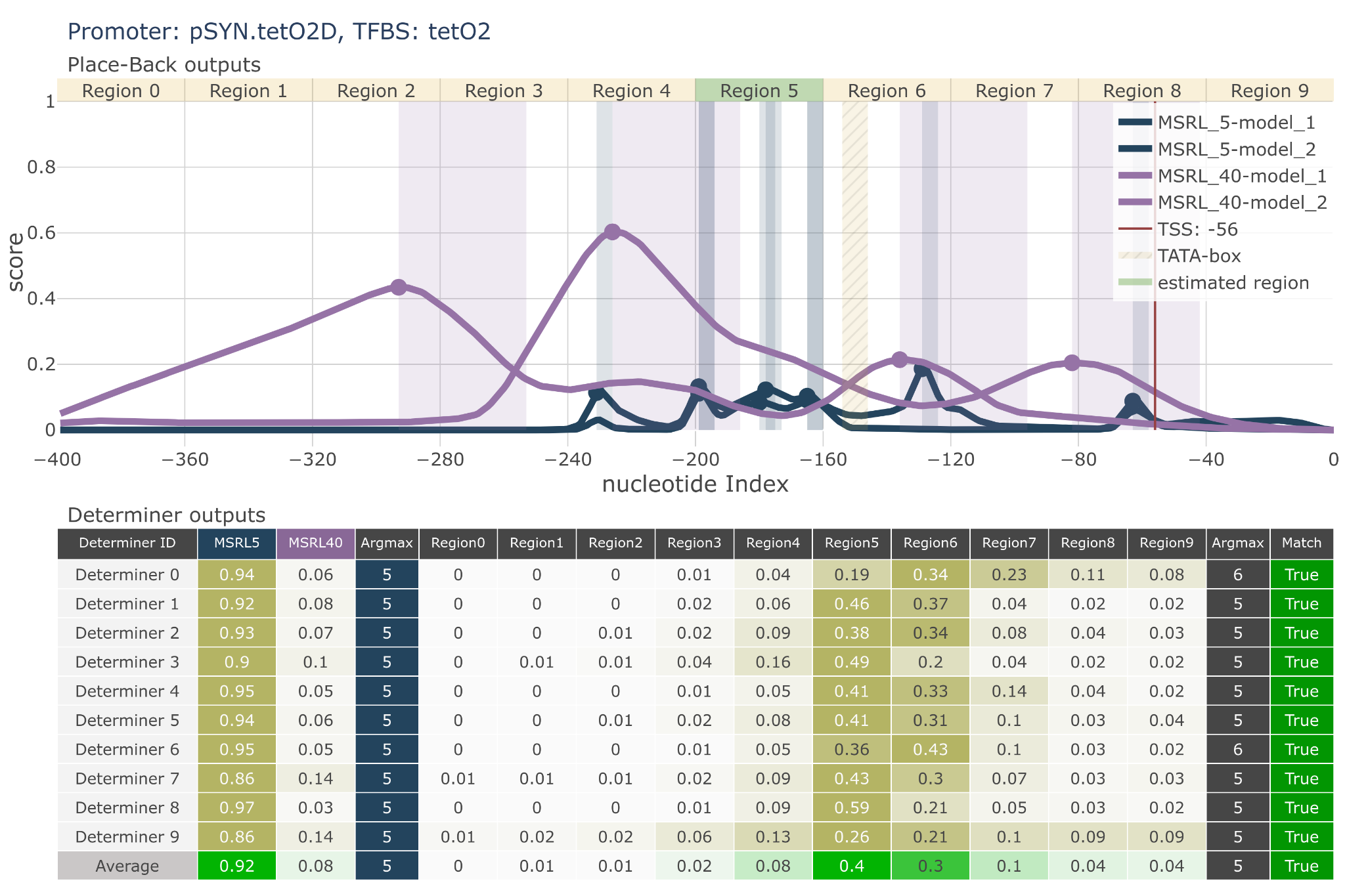


**Supplementary Figure 19**: Complete output from the two-stage ANN system for the second-round recombination of the Psyn.tetO2D-like promoter (derived from Supplementary Fig. 18) with tetO2 [19]. The primary model decision suggests either rewriting the tetO2 inserted in the first round or inserting a new site at -199 to -194 (mimicking Psyn.tetO2CD [19], an untested combination). Alternative second-round 5 bp predictions include: (1) -231 to -226 (mimicking the untested Psyn.tetO2BD [19]); (2) -129 to -124 (a construct between the tested Psyn.tetO2Da and Psyn.tetO2Db [19]); and (3) -62 to -58 (mimicking Psyn.tetO2Dc [19], which exhibited the second-strongest repression for this promoter-TFBS combination).


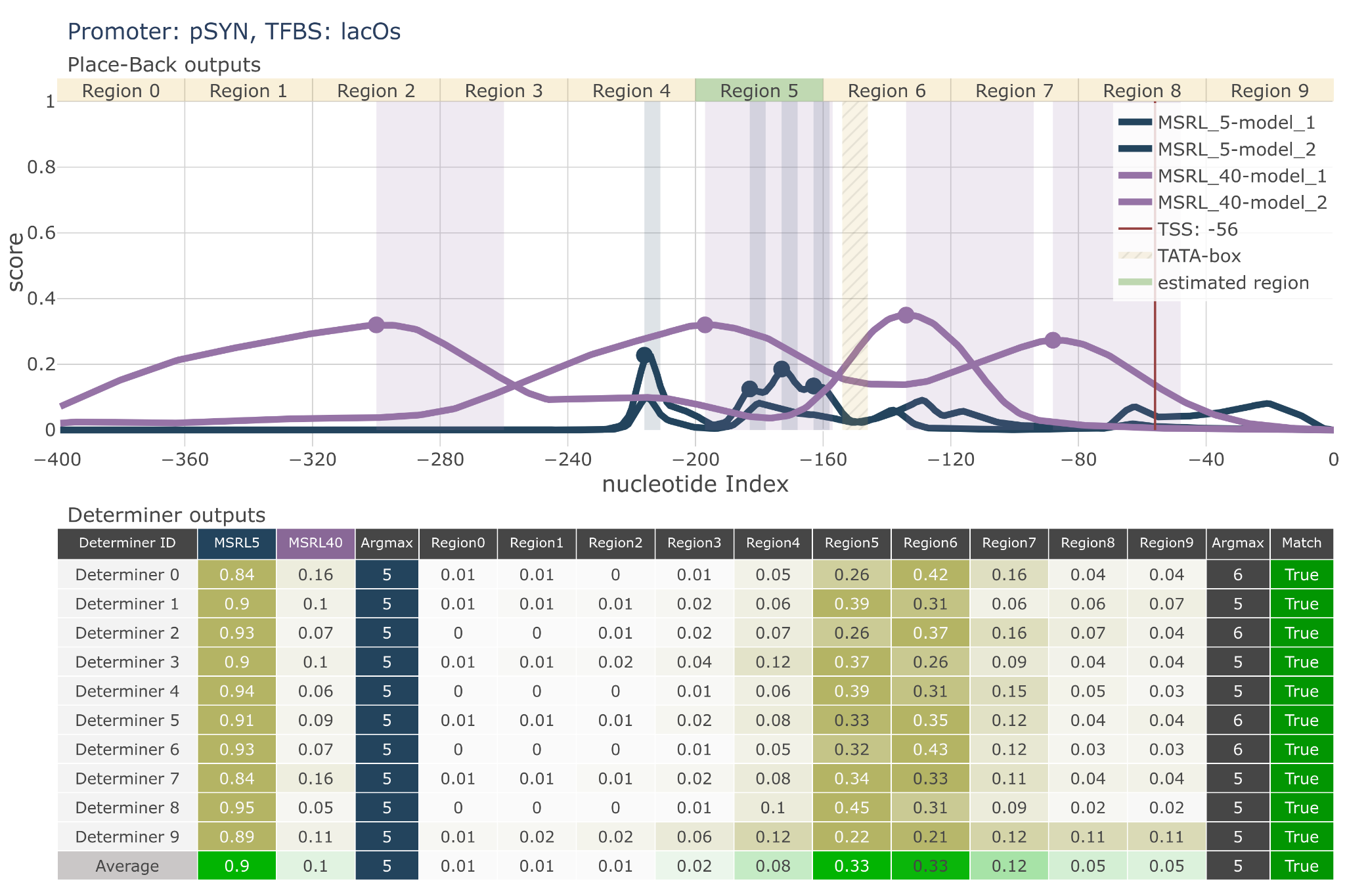


**Supplementary Figure 20**: Complete output from the two-stage ANN system for the recombination of the pSYN promoter [19] with lacOs [19]. The selected lacOs placement is from -173 to -168, mimicking the Psyn.lacOsD construct from [19]. This first-round modification (single-site lacOs) serves as the input promoter for the second round of design (see Supplementary Fig. 21).


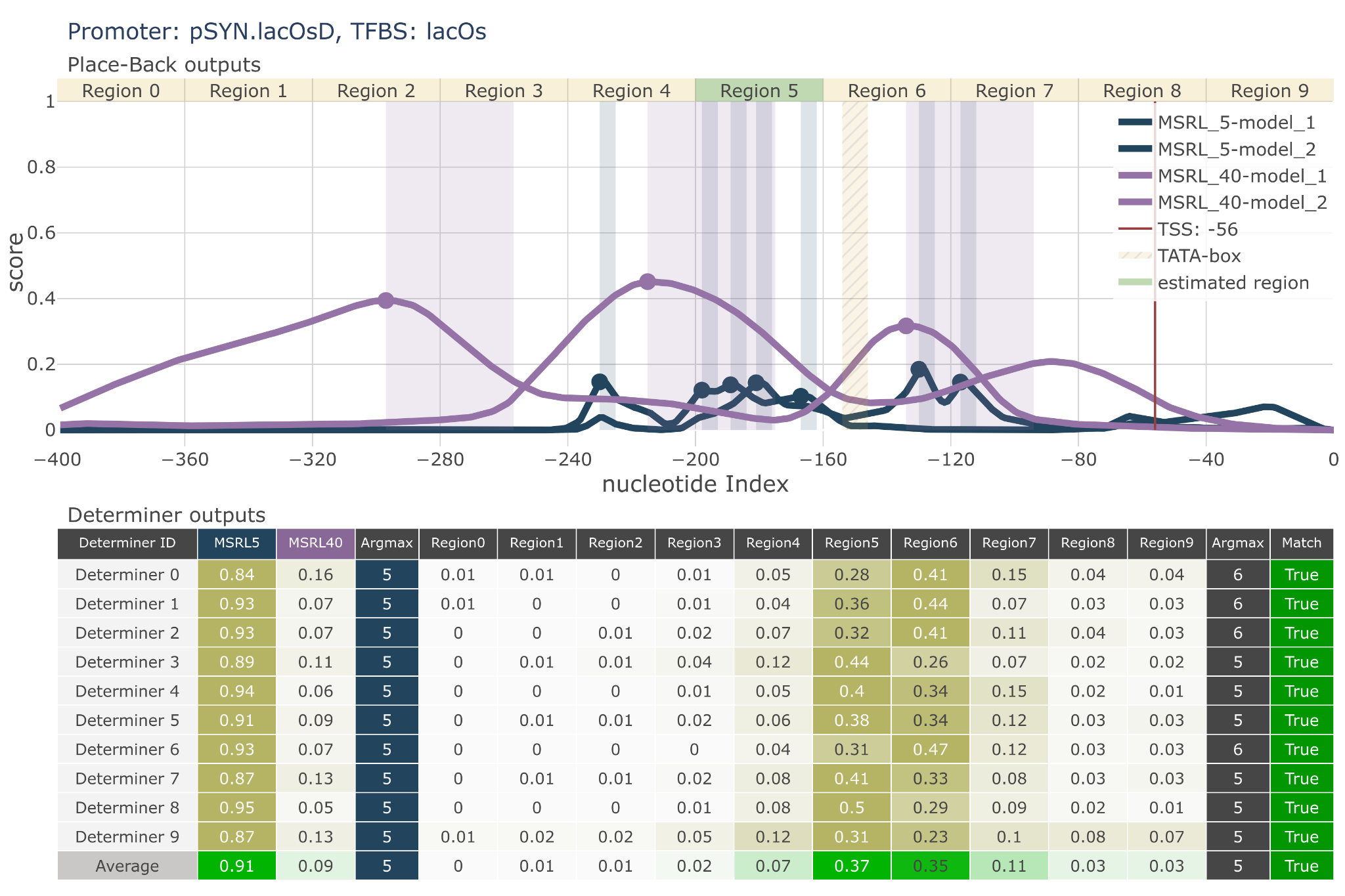


**Supplementary Figure 21**: Complete output from the two-stage ANN system for the second-round recombination of the Psyn.lacOsD-like promoter (derived from Supplementary Fig. 20) with lacOs [19]. The primary model decision suggests either rewriting the lacOs inserted in the first round or inserting a new site at -198 to -193 (mimicking Psyn.lacOsCD [19], an untested combination). Alternative second-round 5 bp predictions include: (1) -230 to -225 (mimicking the untested Psyn.lacOsBD [19]); (2) -130 to -125 (mimicking Psyn.lacOsDa [19], which showed moderate repression); and (3) -117 to -112 (mimicking Psyn.lacOsDb [19], which exhibited the fourth-strongest repression for this promoter-TFBS combination).

**Tables**

| **Promoter** | **Version** | **Repression** | **Disruption Rate** | **Insertion** | **Outcome** |
| --- | --- | --- | --- | --- | --- |
| **pHHF2** | Alternative | 14.2% | -7% | -177 to -137 | Weak Repression |
|  | **Model** | **35.8%** | **-6%** | **-235 to -195** | **Improved Repression** |
| **pPAB1** | Alternative | 88.5% | 28% | -195 to -145 | Functional |
|  | **Model** | **90.1%** | **55%** | **-278 to -238** | **Similar Repression** |
| **pPOP6** | Alternative | -199.1% | 93% | -161 to -121 | Broken (Dead Promoter) |
|  | **Model** | **98.4%** | **19%** | **-70 to -65** | **High Performance** |
| **pREV1** | Alternative | -141.5% | 91% | -116 to -76 | Broken (Dead Promoter) |
|  | **Model** | **62.7%** | **70%** | **-277 to -237** | **Functional** |
| **pFIG1** | **Model** | **0.0%** | **99%** | **-238 to -198** | **Correctly Predicted Incompatible** |

**Supplementary Table 1:** **Comparison of functional metrics between the Model Recommendation and Alternative Sites for tetO insertions.** This table contrasts the Repression Rate (efficiency of the OFF state) and Disruption Rate (loss of basal activity relative to wild-type) for promoters designed by the reported ANN model versus alternative insertion sites within the same promoters that were not proposed by the model. The Alternative Sites serve as a proxy for suboptimal or semi-random insertion placements. Key observations: (1) Alternative Sites frequently resulted in broken regulatory logic (indicated by negative repression rates in Alternative pPOP6 and pREV1) or severe loss of function (e.g., Alternative pPOP6 and pREV1 showing >90% disruption), confirming that tetO insertion is not universally tolerated. (2) The Model Recommendation consistently identified functional insertion sites, restoring regulatory logic (positive repression) and significantly reducing disruption in sensitive promoters (e.g., pPOP6 disruption reduced from 93% to 19%). (3) pFIG1 was explicitly flagged by the model as "incompatible" and is included as a negative control; experimental testing confirmed it as non-functional, supporting the model's discriminative power.
